# Digital Display Precision Predictor: the prototype of a global biomarker model to guide treatments with targeted therapy and predict progression-free survival

**DOI:** 10.1038/s41698-021-00171-6

**Published:** 2021-04-28

**Authors:** Vladimir Lazar, Shai Magidi, Nicolas Girard, Alexia Savignoni, Jean-François Martini, Giorgio Massimini, Catherine Bresson, Raanan Berger, Amir Onn, Jacques Raynaud, Fanny Wunder, Ioana Berindan-Neagoe, Marina Sekacheva, Irene Braña, Josep Tabernero, Enriqueta Felip, Angel Porgador, Claudia Kleinman, Gerald Batist, Benjamin Solomon, Apostolia Maria Tsimberidou, Jean-Charles Soria, Eitan Rubin, Razelle Kurzrock, Richard L. Schilsky

**Affiliations:** 1 Worldwide Innovative Network (WIN) Association - WIN Consortium, Villejuif, France; 2 Institut Curie, Paris, France; 3 Pfizer Inc., San Diego, CA USA; 4 Merck KGaA, Darmstadt, Germany; 5 Sheba Medical Center, Tel-Hashomer, Israel; 6 ARC Foundation for cancer research, Villejuif, France; 7 Iuliu Hatieganu University of Medicine and Pharmacy, Cluj-Napoca, Romania; 8 The Oncology Institute “Prof. Dr. Ion Chiricuta”, Cluj-Napoca, Romania; 9 I.M Sechenov First Medical State University, Moscow, Russian Federation; 10 Vall d’Hebron Hospital Campus and Institute of Oncology (VHIO), IOB-Quiron, UVic-UCC, Barcelona, Spain; 11 Ben-Gurion University of the Negev, Beer-Sheeva, Israel; 12 Segal Cancer Centre, Jewish General Hospital, McGill University, Montréal, and NCE Exactis Innovations, Montreal, QC Canada; 13 Avera Cancer Center, Sioux Falls, SD USA; 14 University of Texas M.D. Anderson Cancer Center, Houston, TX USA; 15 Gustave Roussy, Villejuif, and University Paris-Saclay, Orsay, France; 16 University of California San Diego, Moores Cancer Center, San Diego, CA USA; 17 American Society of Clinical Oncology (ASCO), Alexandria, VA USA

**Keywords:** Computational biology and bioinformatics, Predictive markers, Oncology

## Abstract

The expanding targeted therapy landscape requires combinatorial biomarkers for patient stratification and treatment selection. This requires simultaneous exploration of multiple genes of relevant networks to account for the complexity of mechanisms that govern drug sensitivity and predict clinical outcomes. We present the algorithm, Digital Display Precision Predictor (DDPP), aiming to identify transcriptomic predictors of treatment outcome. For example, 17 and 13 key genes were derived from the literature by their association with MTOR and angiogenesis pathways, respectively, and their expression in tumor versus normal tissues was associated with the progression-free survival (PFS) of patients treated with everolimus or axitinib (respectively) using DDPP. A specific eight-gene set best correlated with PFS in six patients treated with everolimus: *AKT2*, *TSC1*, *FKB-12*, *TSC2*, *RPTOR*, *RHEB*, *PIK3CA*, and *PIK3CB* (*r* = 0.99, *p* = 5.67E−05). A two-gene set best correlated with PFS in five patients treated with axitinib: *KIT* and *KITLG* (*r* = 0.99, *p* = 4.68E−04). Leave-one-out experiments demonstrated significant concordance between observed and DDPP-predicted PFS (*r* = 0.9, *p* = 0.015) for patients treated with everolimus. Notwithstanding the small cohort and pending further prospective validation, the prototype of DDPP offers the potential to transform patients’ treatment selection with a tumor- and treatment-agnostic predictor of outcomes (duration of PFS).

## Introduction

The application of personalized medicine to oncology has resulted in prominent successes that have led to approved, molecularly specific, biomarker-defined indications for targeted therapies. As examples, the use of *EGFR* mutation/erlotinib^1^, *KIT* mutation/imatinib^2^, *BRAF* mutation/vemurafenib^3^, *ALK* translocation/crizotinib^4^, and high tumor mutation burden or microsatellite instability high/pembrolizumab^5–8^, have dramatically changed the treatment landscape in many cancers including, but not limited to melanoma, non-small cell lung carcinoma (NSCLC), colorectal carcinoma (CRC), and head and neck (HN) cancers.

However, despite the advent of personalized precision oncology, cancer remains one of the leading causes of deaths all over the world. Globally, 9.6 million deaths are attributed to cancer, representing 13% of all deaths^9^.

Extending the application of precision medicine requires a deeper understanding of tumor biology. Furthermore, improvement in the ability to select patients is needed, both with respect to identifying sensitive versus resistant tumors and in pinpointing patients at risk for severe toxicities.

With the number of validated drug targets increasing, testing each patient’s tumor for all markers related to all possible targeted therapies becomes infeasible due to the limited amount of tissue usually obtained by biopsies, pointing out the limitation of the classic companion diagnostic approach.

A comprehensive analysis of all relevant genes in a single assay would enable the exploration of all drug targets simultaneously to inform therapeutic options for patients. Furthermore, the complexity of cancer biology requires investigation of multiple genes in networks of pathways to understand the variability of clinical outcomes observed, that cannot be achieved by investigating single genes (as performed with most companion diagnostic tests).

We report the Digital Display Precision Predictor (DDPP), a biomarker strategy and tool, able to predict duration of progression-free survival (PFS) for multiple targeted treatments, based on the comprehensive investigation of the whole transcriptome. The DDPP prototype presented here was derived from analysis of transcriptomic and clinical outcomes in patients with advanced cancer enrolled in the WINTHER clinical trial and treated with: everolimus (mTOR inhibitor), axitinib (VEGFR receptors inhibitor), afatinib (pan-HER inhibitor), trametinib (MEK inhibitor), FGFR inhibitors, and anti-PD-1/PDL-1 antibodies (pembrolizumab, nivolumab and atezolizumab). The WINTHER trial (NCT 01856296) explored, for the first time in a prospective clinical trial, the use of differences in gene expression between tumor and analogous organ-matched normal tissues to guide treatment selection^10^. The trial demonstrated that transcriptomic analysis, based on tumor/normal tissue comparison, was feasible and increased, by about one third, the number of patients that could be matched to a targeted therapy as compared to genomic analysis alone.

## Results

### DDPP strategy and objectives

The objective of the DDPP was to build a predictor of the PFS as a continuous variable, in contrast with current binary predictive biomarkers that predict if patients will respond to a specific therapeutic regimen or not. The DDPP transcriptomic-based biomarker strategy is based on three pillars: (1) gene selection—literature review was used to identify a list of key genes governing the sensitivity for a given therapeutic regimen and the exploration of their differential expression in tumor compared to analogous normal tissues. Comprehensive transcriptomic analysis of the whole transcriptome (~20,000 genes) in a single assay requiring a very small amount of tissue, which enables the exploration of all targets simultaneously to inform therapy options for patients. Reducing the number of features (genes) for consideration reduces the chances of overfitting and allows useful predictions to be made regardless of the size of the patient cohort; (2) ranking each of the genes from the identified list of relevant genes, based on their relative association with PFS. Different conventional ranking methods were considered, including Cox univariate and multivariate regression models, multiple linear regression (MLR), or parametric (Pearson) and nonparametric (Spearman) correlation tests. We selected the Pearson correlation with PFS, since it outperformed all other ranking methods (presented in “Methods” and in Supplemental Notes); and (3) building a PFS predictor. Once the relevant genes were ranked, we applied an empirical summation approach, comparing the performance of every set of genes with the top K genes (K = 1..*N*, where *N* is the number of genes in the list identified in step (1) above) in predicting PFS. For every subset with the top-ranking K genes, four summation methods were used to predict PFS: mean, median, product, and absolute product.

### DDPP investigations of patients treated with everolimus

We applied the DDPP method to build a PFS predictor for everolimus treatment: Supplemental Table 1 describes the currently recognized 17 key genes of the mTOR pathway^11,12^: upstream regulators of *MTOR*: *PIK3CA*, *PIK3CB*, *AKT1*, *AKT2*, *PTEN*, *TSC1*, *TSC2*, *RHEB*, and *FKB-12 (FKBP1A)* plays a key role as it binds to everolimus and interacts with MTOR, resulting in the formation of the inhibitory complex MTORC1 (*MTOR*, *MLST8*, and *RPTOR*) and MTORC2 (*MTOR*, *MLST8*, and *RICTOR*); downstream effectors: S6K1 (*RPS6KB1)*, *4EBP1 (EIF4EBP1)*, *HIF1 (HIF1A)*, and *VEGFA*.

**Fig. 1:**
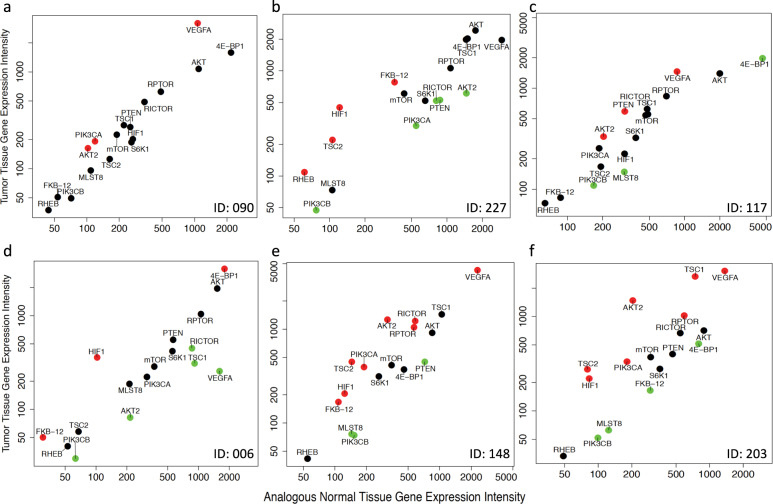
DDPP intensity plots profiles for patients treated with everolimus. DDPP tumor/normal intensity plots profiles of differential gene expression of the everolimus key genes for the six patients treated with everolimus in monotherapy. Clinical outcome is described in Table 1. **a** ID 090, head and neck carcinoma, PFS = 1.3 months (progressive disease), third therapy line. **b** ID 227, liposarcoma, PFS = 1.7 months (progressive disease) in fifth therapy line. **c** ID 117, head and neck carcinoma, PFS = 1.9 months (progressive disease), third therapy line. **d** ID 006, unknown primary origin, PFS = 8.1 months (stable disease) in second therapy line. **e** ID 148, neuroendocrine tumor of small gut PFS = 11.6 months (stable disease) in third therapy line. **f** ID 203, neuroendocrine tumor of small intestine, PFS = 60+ months (partial response disease) in second therapy line. *Y* axis: intensity of the expression in tumors, *X* axis: intensity of the expression in normal matched tissue. Intensities are measured as relative fluorescence unit (RFU) signal as assessed with Agilent microarray technology. Overexpression for a given mRNA in the tumor as compared to the normal is denoted in red points, underexpression is denoted in green, and no change is denoted in black. Data source WINTHER trial^10^.

In six patients treated with everolimus, the fold changes measuring the differential tumor versus normal gene expression of each of the 17 key genes, creates 17 different coordinates defined by the fold changes in tumor and the steady-state levels of mRNAs in tumor and normal tissues. The main clinical and outcome characteristics of the patients treated with everolimus are described in Table 1, together with next-generation sequencing Foundation One test (Foundation Medicine) performed during the WINTHER study. Results of the WINTHER trial have been previously published^10^. One patient treated with everolimus (ID 203) had an exceptional response lasting in excess of 60 months and PFS was therefore censored at this timepoint. It should be noted that the genomic profile could not explain the variation in PFS observed in the everolimus monotherapy group; indeed, the two patients with the longest PFS, both with GI tract neuroendocrine tumors, had no mutations (ID 203) or no genomic alterations in the PI3K/AKT/mTOR pathway (ID 148), respectively. In contrast, the patients with much shorter PFS (ID 227, ID 6, ID 90, and ID 117) did have alterations in the PI3K/AKT/mTOR pathway, albeit accompanied by co-alterations that might have driven resistance^13^. Since DNA biomarkers could not explain variations in clinical outcome, we further investigated whether transcriptomics and DDPP could provide a deeper insight. Figure 1 shows the DDPP profile of the 17 key genes of the six patients treated with everolimus as monotherapy. It should be noted that the DDPP profile is highly variable between the six patients. While *MTOR* has the same steady-state level in both tumor and normal tissues, Fig. 1e, f suggests a trend of greater overexpression of upstream regulators of MTORC1 complex (in particular *TSC1* and *AKT2*) in patients with longer PFS (ID 148 and ID 203), as compared with the patients with shorter PFS (ID 090, ID 227, ID 117, and ID 006).

**Table 1:** Characteristics of the patients treated in WINTHER trial investigated with DDPP.

Study ID	Age	Sex	Cancer site	Prior lines	PFS months	DNA—list of molecular alterations (Foundation Medicine report)^a^	Drug_given
203	67	F	GI/NE	1	60.0+	No mutation	Everolimus
148	82	M	GI/NE	2	11.6	BCOR N1652fs*34; CDKN1B E126fs*1	Everolimus
6	64	F	UP	1	8.1	TSC1 splice site 913 + 1G > T; BRCA1 truncation, intron 11; CDKN2A/B loss; DNMT3A R882H; LRP1B loss	Everolimus
117	34	M	HN	2	1.9	TSC2 S1431L; TP53 G245S; BCOR K374fs*19; SMARCA4 R1135W	Everolimus
227	56	M	LS	4	1.7	STK11 F354L; STK11 F354L; TERT promoter —124C > T	Everolimus
90	74	M	HN	2	1.3	PIK3CA Q546R; EP300 D1154fs*30; NOTCH1 L1746fs*40	Everolimus
83	59	M	HN	4	8.8	MTOR L2209V; ETV6 trunc intron 5; CIC S333fs*36; MLL2 G3698 fs*51	Axitinib
223	65	F	HN	3	7.1	CCND1 T2861	Axitinib
259	53	F	HN	4	6.2	PDGFRA amp	Axitinib
25	65	M	HN	2	5.3	TP53 I195F; KDM6A L725fs*4; MSH6 K1358fs*2; NFE2L2 R18Q	Axitinib
88	56	M	Lung	1	2.9	DNMT3A R635P; KRAS G12C; TP53 Y220C; MLL2 T1246M	Axitinib
149	54	F	CRC	5	7.4	KRAS G12V; ARID1A SPLICE SITE 2733-1G > A	Trametinib
100	43	M	Lung	2	6.6	BRAF A598_T599insT; IDH1 R132C	Trametinib
118	78	F	Lung	3	3.1	KRAS G12C; CDKN2A/B loss; TP53 V157F, Y220 fs*27; MUTYH G382D	Trametinib
156	71	F	Lung	2	14.3	EGFR E746_A750del, T790M; CDKN2A/B loss; CTNNB1 S33F; MYC amplification; SMAD4 P186fs*6; STAG2 splice site 1535-12_1630del108	Afatinib + cetuximab
235	60	F	Lung	1	11.3	ERBB2 A775_G776insYVMA	Afatinib
136	79	M	Lung	3	0.4	ERBB3 amp; MET splice site 3028 + 1G > A; STK11 Q100* ATM L2450fs*11; BRCA1 E23fs*17; CDK4 amp; CDKN2A/B loss; MDM2 amp; APC I1307K; KDM5C truncation; MAP3K1 S1475*	Afatinib
237	47	M	HN	6	19.3	CCND1 amp; FGFR2 amp; CDKN2A/B loss; FGF19 amp; FGF4 amp; BAP1 trunc exon 3; FGF3 amp; MAGI2 Q1077*; PBRM1 E1155fs*17	NCT01004224 BGJ398
247	67	M	Esophagus	2	1.6	FGFR2 amp; CDKN2A/B loss; TP53 W91*; ASXL1 splice site 472-2A > G	NCT02052778 TAS-120
228	38	M	CRC	5	0.7	FGFR1 amp; TP53 C176F; APC E1322*, R213*; SMAD4 loss; SOX9 V163fs*21	NCT02052778 TAS-120
183	66	M	CRC	2	61.0+	RBB3; V104M; MAP2K1; E203K; CDKN2A/B loss; FBXW7 R465C; PIK3CA E39K; PIK3R1 R348*, R639*; PTEN R233*, splice site 801 + 2T > G; TP53 R158H, R273H; APC R1450*, R499*; ARID1A P1115fs*46, Q1306fs*17; ATRX Q2422*; CDH1 D433N; EP300 R2263*; FAM123B R631*; FAT1 A4305V; FLCN H429fs*39; MSH6 L1330fs*12, S279fs*12	Pembrolizumab (TMB: 74.8) (MSI: +)
294	57	M	HN	1	1.7	BRCA2 K3408*	Nivolumab (TMB: 0) (MSI: −)
270	76	F	CRC	3	0.9	FLT4 amp; FLT3 amp equivocal; BARD1 C53fs*5; MYC amp; PARK2 loss exons 3–5; TP53 R175H; APC T1556fs*3; BCL2L1 amp; CDK8 amp; ETV6 rearrangement intron 5; FAM123B R497*; GATA6 amp equivocal; KDM6A-Y215*; MUTYH-Y165C; NOTCH1 Q2123*	Atezolizumab (TMB: 10.4) (MSI: −)

Foundation Medicine^a^^10^; *ID* 203, PFS 60+ and OS60+ are censored values, updated from the WINTHER trial Supplemental Table 3, Abbreviations: *GI* gastrointestinal, *NE* neuroendocrine, *HN* head and neck, *UP* unknown primary, *LS* liposarcoma, *TMB* tumor mutation burden, *MSI* microsatellite instability, *amp* amplification, *del* deletion, *trunc* truncation.

The relative impact on the PFS of each individual gene was determined through Pearson correlations between differential gene expression and PFS for each of the 17 genes were *AKT2* (*r* = 0.75, *p* = 0.087), *TSC1* (*r* = 0.74, *p* = 0.094), *FKB-12* (*r* = −0.67, *p* = 0.149), *TSC2* (*r* = 0.63, *p* = 0.178), *RPTOR* (*r* = 0.61, *p* = 0.198), *RHEB* (*r* = −0.49, *p* = 0.325), *PIK3CA* (*r* = 0.43, *p* = 0.4), *PIK3CB* (*r* = −0.41, *p* = 0.414), *AKT1* (*r* = −0.35, *p* = 0.496), *MLST8* (*r* = −0.34, *p* = 0.509), *VEGFA* (*r* = 0.27, *p* = 0.604), *HIF1* (*r* = 0.27, *p* = 0.606), *PTEN* (*r* = −0.16, *p* = 0.759), *4EBP1* (*r* = −0.16, *p* = 0.77), *RICTOR* (*r* = 0.14, *p* = 0.788), *MTOR* (*r* = 0.13, *p* = 0.807), and *S6K1* (*r* = −0.04, *p* = 0.936). We further explored the combined differential expression in tumor versus normal tissues of the most contributive key genes involved in the everolimus pathway. For each of the correlations with PFS, we built a vectorial summation using a “step-in” method, starting with *AKT2*, which was identified as the most contributive gene according to the Pearson correlations, and adding successively a single gene at each step in the order of their significance: *AKT2-TSC1*; *AKT2-TSC1-FKB12*, then *AKT2-TSC1-FKB12-TSC2* and so forth, obtaining in total 17 different vector summations. Each combined vector was correlated with the observed PFS.

**Fig. 2:**
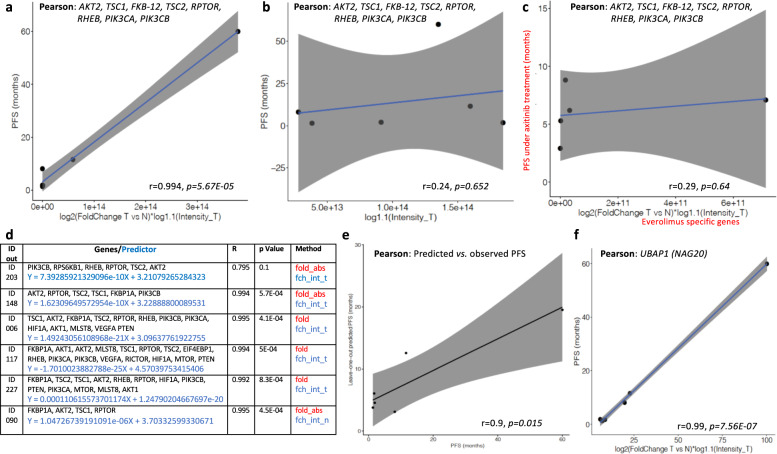
DDPP correlation with PFS for patients treated with everolimus. **a** Pearson correlation plots of the eight-gene predictor: *AKT2*, *TSC1*, *FKB-12*, *TSC2*, *RPTOR*, *RHEB*, *PIK3CA*, and *PIK3CB* with the PFS of six patients treated with everolimus as monotherapy: one patient out of these six had censored PFS (ID 203). *X* axis: absolute fold value of log2 based fold-changes tumor versus normal multiplied by log 1.1 based of the intensities in tumor values for each of the eight genes selected; *Y* axis: PFS under treatment with everolimus as monotherapy in months. **b** Pearson correlation plots of the eight-gene predictor: *AKT2*, *TSC1*, *FKB-12*, *TSC2*, *RPTOR*, *RHEB*, *PIK3CA*, and *PIK3CB* with the PFS of six patients treated with everolimus as monotherapy when only the tumor biopsy is investigated; *X* axis: absolute fold value of log 1.1 based of the intensities in tumor values for each of the eight genes selected; *Y* axis: PFS under treatment with everolimus as monotherapy in months. **c** Shuffle experiment: Pearson correlation plots of the 8 gene specific predictor for everolimus (*AKT2*, *TSC1*, *FKB-12*, *TSC2*, *RPTOR*, *RHEB*, *PIK3CA*, and *PIK3CB)* with the PFS of five patients treated with axitinib as monotherapy (Table 1); *X* axis: absolute fold value of log2 based fold-changes tumor versus normal multiplied by log 1.1 based of the intensities in normal values for each of the eight genes selected; *Y* axis: PFS under treatment with axitinib as monotherapy in months. **d** Leave-one-out experiments: each reiteration generates a predictor used to calculate PFS of the patient discarded. **e** Pearson correlation between leave-one-out predicted PFS and the observed PFS; *X* axis: predicted PFS as defined by leave-one-out (in months); *Y* axis: PFS under treatment with everolimus as monotherapy in months observed in WINTHER trial. **f** Pearson correlation plot for *UBAP1 (NAG20)* with the PFS of six patients treated with everolimus. *X* axis: log2 based fold-changes tumor versus normal multiplied by log 1.1 based of the intensities in tumor; *Y* axis: PFS under treatment with everolimus as monotherapy in months.

Figure 2a shows that the optimal performance was obtained by the vectorial summation of the eight most contributive genes *AKT2*, *TSC1*, *FKB-12*, *TSC2*, *RPTOR*, *RHEB*, *PIK3CA*, and *PIK3CB*, which showed the most significant correlation with PFS, among the 17 possibilities: (*r* = 0.99, *p* = 5.67E−05). The higher the relative expression of these key genes in tumor tissue, the longer the PFS is under treatment with everolimus. The linear regression model for the correlation with PFS is *Y* = 1.499E−13*X* + 3.134 (Equation 1), where *X* = the absolute value of the fold of log2(fold change tumor versus normal) multiplied by log1.1 (Intensity_Tumor) of each value for each of the eight genes, and *Y* = PFS in months. Allelic frequency of mutations was not taken into account, as mutations could not explain variations in PFS. Allelic frequency was not available as transcriptomics was performed with microarray technology. It should be noted that the most contributive genes, *AKT2*, *TSC1*, *FKB-12*, *TSC2*, *RPTOR*, and *RHEB* are key for direct interaction with MTOR and its upstream regulation (TSC1, TSC2, and RHEB). Furthermore, *FKB-12* binds everolimus and associates to MTOR forming together with RPTOR the MTORC1 complex.

Figure 2b shows that when only the tumor biopsy was investigated (as is usually done in the current biomarker studies in oncology practice or translational research), the significance of the correlation dropped to *r* = 0.24, *p* = 0.65, suggesting the critical importance of the new strategy of assessing tumor versus normal analogous organ-matched dual biopsy.

In order to assess the prognostic versus the predictive value of the DDPP data in our analyses, we tested the specific predictor of the PFS for everolimus (*n* = 6 patients) generated by eight genes (*AKT2*, *TSC1*, *FKB-12*, *TSC2*, *RPTOR*, *RHEB*, *PIK3CA*, and *PIK3CB)* and cross-correlated their combined differential expression with the PFS of patients under axitinib treatment (*n* = 5, Table 1). Figure 2c demonstrates that the subset of genes identified for everolimus was highly specific to the everolimus regimen, as the cross correlation with PFS of patients treated with axitinib was not significant (*r* = 0.29, *p* = 0.637), suggesting that the DDPP findings are consistent with the known biology of the *MTOR* pathway and the mechanism of action of everolimus.

To interrogate whether the findings of these analyses could be used as predictors, and address the potential for overfitting, we performed leave-one-out analyses, reiterating six combinatorial analyses. At each investigation, one patient was discarded, and a correlator/predictor was identified based on the remaining five patients applying the same methodology. The correlator was then used as a predictor to predict the PFS of the patient left out. Figure 2d depicts the six correlations obtained in the reiterative experiments, and Fig. 2e shows the correlation between the observed PFS and the leave-one-out predicted PFS. At each reiteration, the predictor is generated by different subsets of genes, suggesting a high impact of the composition of the cohort when small number of patients are investigated.

This observation pinpoints the need to validate the DDPP on a larger cohort of patients. Nevertheless, the concordance between the real PFS of the patients left out, and the predicted PFS using the correlator obtained at each reiteration on the remaining five patients is significant, with *r* = 0.9, *p* = 0.015.

We evaluated the performance of the predictive method by an independent method assessing the root mean squared error (RMSE) that was 16.82. The mean absolute error that measures the magnitude of the error without taking into consideration the direction of the error unlike the RMSE was 9.32. Given the small number *n* = 6, these data suggest a good performance of the predictive model. These preliminary observations suggest the realistic possibility of obtaining a stable predictor, using a higher number of patients, to obtain a validated tool that may be useful to accurately estimate the PFS in a prospective clinical setting.

In order to assess the potential of the DDPP method for biomarker discovery, we performed random selections of eight genes (number corresponding to the optimal number of genes of the specific everolimus predictor) across the whole transcriptome (~20,000 genes), and correlated their vectorial summation with PFS of the six patients who received single agent everolimus treatment. This analysis was repeated 100,000 times, randomly selecting a different set of eight genes at each reiteration. Setting the threshold of significance at the same value as the one observed for the predictor (*r* = 0.99, *p* = 5.67E−05), the percentage of random significant correlations with PFS was 0.994%. The observation that randomly selected sets of genes generated significant correlations with PFS suggests that (i) specificity of correlations could be increased only with a larger number of patients used as training and test datasets, and (ii) DDPP could potentially be used for identification of new genes/biomarkers, if used not in a random selection manner, but rather in a systematic selection manner.

#### DDPP tested as a biomarker discovery method

The most significant correlation between the differential expression of a new identified gene and PFS of patients treated with everolimus was observed for *UBAP1* (ubiquitin associated protein 1): *r* = 0.99, *p* = 7.56E–07 (Fig. 2f). *UBAP1 (NAG20)* is a component of the ESCRT-I complex, a regulator of vesicular trafficking that could be potentially involved in degradation of MTORC2 and directly linked to MTOR pathway^14^.

### DDPP and prediction of outcome with axitinib

At nanomolar concentrations, axitinib specifically inhibits VEGFR1, VEGFR2, and VEGFR3. Thirteen key genes involved in the control of angiogenesis were selected and investigated with DDPP methodology: *FLT1 (VEGFR1)*, *KDR (VEGFR2)*, *FLT4 (VEGFR3)*, and their ligands *VEGFA*, *VEGFB*, *VEGFC*, and *FIGF*, *PDGFRA*, *PDGFRB*, *PDGFA*, *PDGFB*, *KIT*, and *KITLG*^15,16^. Four patients had HN carcinoma, and one patient had a lung adenocarcinoma. Table 1 shows the different PFS under treatment with axitinib observed in the WINTHER trial.

The differential tumor versus normal expression of *KIT* and of its ligand *KITLG* was identified as being the major driver of the correlation with the PFS of the patients treated with axitinib (Supplemental Fig. 1a, b), and their combined vector *KIT-KITLG* generated the optimal performance in the correlation with PFS: *r* = 0.99, *p* = 4.68E−04 (Supplemental Fig. 1c). The linear regression model is *Y* = 2.014e−02*X* + 4.36 (Equation 2), where *X* = the sum of log2(fold change tumor versus normal) multiplied by log1.1 (Intensity_Normal) of each value for each of the two genes, and *Y* = PFS in months).

Random selections of two genes across the whole transcriptome and correlation of their vectorial summation with PFS of the five patients treated with axitinib was repeated 100,000 times. Using the same threshold as the specific predictor (*r* = 0.99, *p* = 4.68E−04) the percentage of significant correlations was 0.059%.

#### Leave one out experiments

We performed (using the same “step-in” vectorial summation methodology) five leave-one-out reiterations, discarding at each experiment one patient and building a predictor on the remaining four to address potential overfitting. We observed again an instability of the predictors and dependence on the composition of the cohorts at each reiteration. The concordance between real PFS of the patients left out, and the predicted PFS using the correlator obtained at each reiteration was lower than for the everolimus example (*r* = −0.81, *p* = 0.1) likely related to a lower number of patients in each reiteration. These data suggest again that performance and accuracy of the prediction of the PFS could be increased only with a higher number of patients in the training and validation datasets.

### DDPP and other examples of TKIs PFS predictors

#### Trametinib

Thirteen key genes were investigated: *MEK1* (*MAP2K1)*, *MEK2 (MAP2K2)*, *ARAF*, *BRAF*, *RAF1*, *ERK1 (MAPK3)*, *ERK2 (MAPK1)*, *MAPK10*, *KRAS*, *HRAS*, *NRAS*, *KSR1*, and *RAP1A*. The combined differential tumor versus normal tissue expression of nine genes and their vectorial summation correlated with the PFS of three patients treated with trametinib as monotherapy (Table 1 and Supplemental Fig. 2a–c)*: ERK2*, *ARAF*, *CRAF*, *MEK1*, *MEK2*, *HRAS*, *ERK1*, *MAPK10*, and *KSR1* (*r* = −0.99, *p* = 0.026); the linear regression model is *Y* = −6.872e−15*X* + 7.745 (Equation 3), where *X* = the fold of log2(fold change tumor versus normal) multiplied by log1.1 (Intensity_Tumor) of each values for each of the nine genes, and *Y* = PFS in months.

#### Afatinib

Thirteen key genes were investigated: *EGFR*, *ERBB2*, *ERBB3*, *ERBB4* and their ligands *EGF*, *TGFA*, *AREG*, *EREG*, *HBEGF*, *BTC*, *NRG1*, *NRG2*, *and NRG4*. The combined differential tumor versus normal tissue expression of two genes and their vectorial summation correlated with the PFS of three patients treated with afatinib as monotherapy (Table 1 and Supplemental Fig. 2d–f): *NRG4 and NRG2* (*r* = −1 *p* = 8.4E−04). The linear regression model is *Y* = −4.558e−02*X* + 2.549 (Equation 4), where *X* = the sum of log2(fold change tumor versus normal) multiplied by log1.1 (Intensity_Tumor) of each value for each of the two genes, and *Y* = PFS in months.

#### FGFR inhibitors

Nineteen key genes investigated: *FGFR1*, *FGFR2*, *FGFR3*, *FGFR4*, and the *FGF* ligands 1, 2, 3, 4, 5, etc.). The differential expression and vectorial summation of five genes correlated with the PFS of three patients treated with FGFR inhibitors BGJ398 or TAS-120 as monotherapy (Table 1 and Supplemental Fig. 2g–i): *FGF10*, *FGF16*, *FGF5*, *FGF2*, and *FGF13* (*r* = −1*,* *p* = 3.27E−03). The linear regression model is *Y* = −5.273e−02*X* + 5.135 (Equation 5), where *X* = the sum of log2(fold change tumor versus normal) multiplied by log1.1 (Intensity_Normal) of each value for each of the five genes, and *Y* = PFS in months.

### DDPP and prediction of outcome after IO treatment

Although the most advanced knowledge has been generated around the therapies targeting PD-1/PDL-1 or CTLA-4, there are multiple other important pathways that may impact the immune response to cancer involving many genes, in particular LAG-3, TLR-4, VISTA, TIM-3, TIGIT, ICOS, OX40, and GITR^5^. Among them LAG-3 and TLR-4 may be particularly important, as described in Supplemental Table 2. Given the current status of knowledge, the IO-specific DDPP gene set focuses on *PDL-1*, *PDL-2*, *PD-1*, *CTLA-4*, *CD28*, *CD80*, *CD86*, *LAG-3*, *and TLR-4*, together with specific markers of the presence of effector tumor-infiltrating immune cells: *CD8A* (cytotoxic lymphocytes T), *CD16* (natural killer cells), and *FOXP3* (T-regs cells). Many types of immune cells are involved in the activation and regulation of the immune system attack against tumor cells (APC, LyT, CD4+, etc.), but we focused on specific markers for infiltrating LyTc, NK, and T-regs that have the ability to recognize directly the tumor cells’ neoantigens coupled with major histocompatibility complex 1 (CMH1) and are directly targeting tumor cells.

**Fig. 3:**
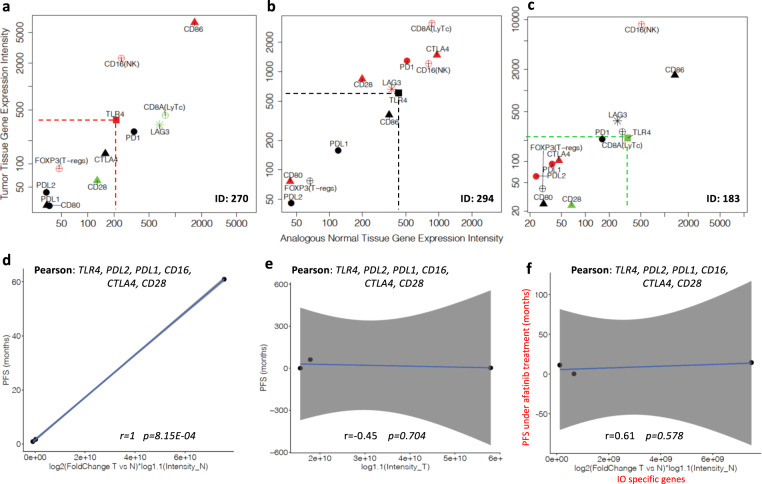
DDPP intensity plots and correlation with PFS for patients treated with anti-PD-1 therapies. **a**–**c** DDPP profiles of three patients treated with anti-PDL-1/PD-1 antibodies, with different PFS under treatment. Data source WINTHER trial^11^. **a** colon cancer: PFS = 0.9 months under treatment with atezolizumab, in fourth therapy line; **b** head and neck cancer: PFS = 1.7 months under treatment with nivolumab in second therapy line; **c** colon cancer: PFS = 61+ months under treatment with pembrolizumab, in third therapy line; *Y* axis: intensity of the expression in tumors; *X* axis: intensity of the expression in normal matched tissue. **d** Pearson correlation between a vectorial summation of 6 genes: *TLR-4*, *PDL-2*, *PDL-1*, *CD16* (specific marker of NK), *CTLA-4,* and *CD28;* *X* axis: fold value of log2(fold change tumor versus normal) multiplied by intensity in normal values for each of the six genes selected; *Y* axis: PFS in months. **e** Pearson correlation between the vectorial summation of the six genes specific anti-PD-1: *TLR-4*, *PDL-2*, *PDL-1*, *CD16* (specific marker of NK), *CTLA-4,* and *CD28*, and the PFS of three patients treated with IO when only tumor biopsy information is investigated; *X* axis: fold value of log2(intensity in tumor) values for each of the six genes selected; *Y* axis: PFS in months. **f** Shuffle experiment: Pearson correlation between the vectorial summation of the six genes specific anti-PD-1: *TLR-4*, *PDL-2*, *PDL-1*, *CD16* (specific marker of NK), *CTLA-4*, and *CD28*, and the PFS of three patients treated with afatinib; *X* axis: fold value of log2(fold change tumor versus normal) multiplied by log 1.1(intensity in normal) values for each of the six genes selected; *Y* axis: PFS of three patients under afatinib treatment (months).

A Pearson correlation analysis between differential gene expression of the selected genes, and the PFS was performed for the three patients treated with anti-PD-1 antibodies (Table 1) in the WINTHER trial. The example provided in Fig. 3a–c shows the DDPP intensity plots of three patients treated with IO (pembrolizumab (anti-PD-1), atezolizumab (anti-PDL-1), and nivolumab (anti-PD-1)); two patients had colon cancer (CRC) and one HN cancer. Their clinical outcomes were ID 183 (CRC) PFS 61+ months (the patient is in complete clinical remission and no longer receiving pembrolizumab), ID 294 (HN) PFS 1.7 months, and ID 270 (CRC) PFS 0.9 months. DDPP profiling identified that the higher the level of *TLR-4* fold change in tumor versus normal tissues, the shorter the survival under treatment with anti-PD-1. Considering that all three treatments are directed to leverage the PDL-1/PD-1 negative blockade, and considering that the mechanisms may be agnostic of tumor type, we performed the investigations aiming to explain the variations in PFS. The relative contribution of each of the key 12 genes was evaluated by correlating their differential expression with the PFS in patients treated with IO. Pearson correlations between differential gene expression and PFS for each of the 17 genes were: *TLR-4* (*r* = −0.99, *p* = *0*.103), *PDL-2* (*r* = 0.97, *p* = 0.143), *PDL-1* (*r* = 0.90, *p* = 0.294), *CD16 (NK)* (*r* = 0.77, *p* = 0.445), *CTLA-4* (*r* = 0.60, *p* = 0.588), *CD28* (*r* = −0.50, *p* = 0.665), *CD80* (*r* = −0.49, *p* = 0.67), *CD86* (*r* = −0.42, *p* = 0.721), *LAG-3* (*r* = 0.34, *p* = 0.776), *CD8A (LyTCD8*+*)* (*r* = −0.30, *p* = 0.803), *FOXOP3 (T-regs)* (*r* = −0.21, *p* = 0.862), and *PD-1* (*r* = −0.18, *p* = 0.882).

We further explored the combined differential expression in tumor versus normal tissues of the most contributive key genes involved in the IO pathway. For each of the correlations with PFS, we built a vectorial summation using a “step-in” method, starting with *TLR-4* and adding successively a gene in the order of their significance: *TLR-4-PDL-2*, *TLR-4-PDL-2-PDL-1*, then *TLR-4-PD-L2-PDL-1-CD16* and so forth, obtaining in total 12 different vector summations. Each combined vector was correlated with PFS. Figure 3d shows that the optimal performance was obtained by the summation of the six most contributive genes: *TLR-4*, *PDL-2*, *PDL-1*, *CD16 (NK)*, *CTLA-4*, and *CD28*, to obtain the most significant correlation with PFS, among the 12 possibilities: (*r* = 1, *p* = 8.15E−04). The linear regression model for the correlation with PFS is *Y* = 7.856e−10*X* – 1.583 (Equation 6), where *X* = the value of the fold of log2(fold change tumor versus normal) multiplied by log1.1 (Intensity_Normal) of each value for each of the six genes, and *Y* = PFS in months.

These data suggest that the main factors explaining differences in PFS under anti-PD-1 therapy are the degree of activation of TLR-4, and the balance between PDL-1, PDL-2, and CTLA-4 activation of the negative immune-blockade. These results might be specific to this cohort (two colon and HN tumors). Figure 3e shows that when only the tumor biopsy was investigated (as usually in the current biomarker studies in oncology practice or translational research), the significance of the correlation dropped to *r* = −0.45, *p* = 0.704, suggesting the importance of the new strategy of tumor versus normal analogous organ-matched dual biopsy.

In order to assess the prognostic versus the predictive value of the DDPP data for IO in our analyses, we tested the specific predictor of the PFS (with the six genes (*TLR-4*, *PDL-2*, *PDL-1*, *CD16 (NK)*, *CTLA-4*, and *CD28*)) for anti-PD-1 treatments (*n* = 3 patients) and cross-correlated their combined differential expression with the PFS of patients under afatinib treatment (*n* = 3, Table 1). Figure 3f demonstrates that the subset of genes identified for anti-PD-1 did not correlate with the PFS of the patients treated with afatinib (*r* = 0.61, *p* = 0.578), suggesting high specificity for IO regimen. Further random selections of six genes (number corresponding to the optimal number of genes of the specific anti-PD-1 predictor) across the whole transcriptome (~20,000 genes), and correlation of their DDPP vectorial summation with the PFS of the three patients who were under anti-PD-1 treatment was repeated 100,000 times, randomly selecting a different set of six genes at each reiteration. Setting the threshold of significance at the same value as the one observed for the predictor (*r* = 1, *p* = 8.15E−04), the percentage of random significant correlations with PFS was 0.356%.

Based on the six genes identified, we assessed “in silico” the predicted PFS of the 82 patients from the WINTHER trial (for whom no information was missing), agnostic of tumor type and independent of the number of prior lines of therapy, if they were treated with anti-PD-1 therapies. For 57 patients (59.5%), the predicted PFS under anti-PD-1 treatment was ≤6 months (with a majority <3 months); for 25 patients (30.5%) the predicted PFS under anti-PD-1 treatment was ≥6 months (of which 16 (19.5%) had predicted PFS > 24 months).

## Discussion

The expanded targeted and immune-oncology therapeutic landscape has revolutionized cancer care, as well as clinical drug development and requires the development of new combinatorial biomarker strategies to overcome the complexity of cancer, improve patients’ stratification and better individualize treatment selection.

Indeed, the investigation of networks of genes would contribute to improving the selection of drugs targeting major hubs that may collapse a network of dysregulated genes^17,18^. Companion diagnostic (CDx) tests currently used in oncology practice to select therapies^19^ (e.g., used to detect specific DNA aberrations), have three main limitations: (1) CDx tests usually investigate single genes, outside of the context of pathways: for example, sensitizing mutations of *EGFR* provide an indication for the use of EGFR inhibitors, such as erlotinib^1^, however the presence of a sensitizing mutation does not necessarily predict response to the treatment^20,21^; (2) the increasing number of drug targets requires testing of multiple genes that quickly becomes unfeasible given the small amount of tissue typically obtained by needle biopsies; and (3) they provide a binary prediction whether patients might respond or not to a specific therapeutic regimen.

In an attempt to overcome the limitations of current oncology biomarkers, our transcriptomic approach allows (1) the exploration of the whole transcriptome (~20,000 genes), providing insight about the status of activation of almost all drug targets in the context of the network of genes or pathways; (2) the data can be obtained from a single assessment requiring very small amount of tumor and analogous normal tissues; and (3) the prediction of the duration of the PFS under a specific therapeutic regimen, as a continuous variable in contrast with binary predictions.

One of the main challenges of finding such new biomarkers is the small size of the available datasets, often involving fewer than 10 patients treated with the same drug, for whom both molecular profiles (from tumor and analogous normal tissues) and PFS data are available.

The work presented here overcomes the above mentioned limitations of current biomarker strategies and identifies the genes and generates linear regression that correlate with clinical outcome. In particular, the specific contribution of the DDPP and the features that distinguish it from other existing methods such as MLR or multiple Cox regression (MCR) consists in the use of nonparametric combination of the relative expression of multiple genes. Indeed, MLR and MCR investigate in the same model not only expression data, but also apply simultaneously coefficients to better differentiate genes impacting outcomes, compared to those that do not. The learning process defining such coefficients is a major limiting factor to these methodologies when using small datasets. DDPP on the other hand, in order to overcome this limitation empirically decides (1) how many features (genes) to include in the predictors rather than stopping when the fit is no longer improved, and (2) which basic parameter-free summation method best predicts the outcome. The final result of the DDPP model consists in defining the equations that link gene expression features to clinical outcome.

However, it is important to emphasize the limitation of the linear regression equations presented here, which are based on a prototype version of the DDPP. Indeed, the DDPP tools presented have the purpose of illustrating the concept and will require validation in a larger prospectively obtained dataset to confirm whether its empirical approach and applicability in any cohort size can be confirmed. Prospective studies will indeed assess the ability of DDPP algorithm to test multiple summation methods in cohorts of different sizes.

One of the pillars, and potential limitations of the DDPP methodology is the investigation of tumor and analogous organ-matched normal tissue biopsies from the same patient, which is not the current practice in standard of care or in translational research and clinical trials. This raises the legitimate question of what is the likelihood of these findings to apply to routine oncology practice. This will be possible only if the DDPP predictor will demonstrate superiority to existing/current biomarkers models and provide a direct benefit for patients.

Use of matched tumor and normal biopsies is essential for accurate interpretation of the transcriptomic data, as it discards the transcriptomic genetic variability background noise in each patient, and lowers significantly the variance of transcriptomic measurements^22,23^. We were able to use such transcriptomic and PFS data from the WINTHER trial database. The WINTHER trial remains the only clinical trial that used transcriptomics in a prospective clinical setting in addition to conventional DNA sequencing to help inform the treatment decision for patients with advanced cancer. WINTHER is also the first and only trial that used the dual biopsy strategy and proved its feasibility and safety, investigating both tumor and analogous normal tissue from the same patient, across a variety of solid tumors^10^. The importance of obtaining good quality tumor and normal tissue biopsies enabling accurate transcriptomic investigations should be noted. All the biopsies used in this study passed stringent histology and RNA quality controls^10^.

With the small cohort available, we explored the DDPP to assess correlations with PFS associated with the drugs everolimus, axitinib, trametinib, afatinib, experimental FGFR inhibitors, and anti-PD-1/PDL-1 therapies received by patients in the WINTHER trial. Remarkably, for all drugs tested, DDPP enabled identification of significant correlations between the differential expression of subsets of key genes and the PFS for each drug investigated. Our preliminary observations show that the DDPP biomarkers seem to be specific to the therapeutic regimens. It should be noted that the DDPP was agnostic of tumor type and independent of the number of prior lines of therapy and could also provide important insight in better understanding the clinical outcomes by identifying the genes with the highest contributing weight driving the correlations. Random testing performed for all drugs and in particular the data obtained from the leave-one-out experiments performed for everolimus, suggest that DDPP predictions are not likely to be statistical artefacts. Moreover, our data suggest that the subsets of genes selected, and the correlations obtained by their combined differential tumor versus normal tissues expression (vector summation) with the PFS for each therapeutic regimen may be specific for each drug and have a predictive value rather than a prognostic value, although this requires confirmation in larger studies.

Taken together, the data suggest the possibility that using a larger number of patients will allow us to generate a validated tool that may be useful to estimate with accuracy the PFS in a prospective clinical setting. Indeed, many drugs investigated in this report, have a narrow spectrum of approved clinical uses, given the prevalence of their pathways in tumor growth and spread, and the reason is probably related to the lack of reliable biomarkers to select patients who might have a therapeutic benefit.

To our knowledge potential biomarkers based on transcriptomics do not exist for the clinical use of everolimus^11,20^ or axitinib^17,24–26^. Indeed, DDPP may provide a methodology and tools that would enable prediction of PFS for any drug (IO, or non-IO targeted therapeutics) or tumor type and in any therapy line. DDPP predictors could be used (pending further validation) to identify the patients who could have clinical benefit from the treatment with everolimus and axitinib that was not predicted by genomic alterations in the WINTHER trial^10^.

The DDPP concept and methodology was tested also on other drugs: trametinib, afatinib, and two experimental FGFR inhibitors (BGJ398 and TAS-120), with a similar mechanism of action. However, the number of patients treated with each of these drugs (*n* = 3), limits our ability to draw firm conclusions and the results are presented only to exemplify that DDPP concept and methodology may apply to any drug, and that similar trends were obtained as in the everolimus and axitinib examples.

We investigated a small cohort (*n* = 3) of patients that received anti-PDL-1 therapies. Preliminary observations suggest that the main factors explaining differences in PFS under anti-PD-1 therapy are the degree of activation of TLR-4, and the balance between PDL-1, PDL-2, and CTLA-4 activation of the negative immune-blockade, together with the level of infiltration of the tumor by natural killer cells. Both DDPP intensity plots and vectorial summation correlative analyses identified TLR-4 as the most contributive gene to explain variations in PFS. Our preliminary observation suggests that the current panel of biomarkers used in clinical practice (tumor mutation burden, microsatellite instability, and PDL-1 status) could be complemented with other potential biomarkers, such as TLR-4. Persistent activation of TLR-4-induced inflammatory signaling in chronic inflammatory conditions can also contribute to carcinogenesis^27^. Experimental evidence suggests association of anti-TLR-4 with anti-PDL-1 treatments could be of interest with the aim to increase the fraction of patients who could benefit from IO treatments.

Validation of DDPP will require further prospective investigation, ideally using biospecimens and clinical data obtained from patients participating in prospective clinical trials^28^, in which implementation of dual tumor and normal biopsy and integration of transcriptomics are feasible^10^.

In conclusion, the clinical records available from the WINTHER trial, and the unique transcriptomic dataset obtained from tumor and organ-matched normal tissue biopsies were essential to enable correlations with clinical outcome under treatment, with TKI inhibitors and IO. The DDPP is potentially a new global biomarker model that can apply to any type of drug (IO or non-IO targeted drugs) alone or in combination, agnostic of tumor type, and can lead, pending further prospective validation, to a new approach to optimal treatment selection for patients with cancer.

## Methods

### Dataset

The DDPP prototype was developed exploiting the transcriptomics data from the WINTHER trial (NCT 01856296—approved in France (ANSM), Spain (Agencia Espanola de Medicamentos y productos sanitarios), Israel (Ministry of Health), Canada (Health Canada), and USA (FDA)). The full methodology of transcriptomic assessment and patient treatment is described in the WINTHER trial published in Nature Medicine^10^. Detailed clinical and biological information for each patient selected for this study is available in Table 1.

Patients who had provided written consent underwent a dual biopsy from the metastasis and from the histologically matched normal tissue. The interventional radiologist selected for biopsy one of the non-necrotic and >2 cm metastatic lesions. The image-guided biopsies were performed under standard operating rules and guidelines using adequate devices, such as trucut 18 gauges needles placed at the periphery of the lesion biopsied. For example, in the case of a liver metastasis biopsy from a rectal adenocarcinoma, the matched normal tissue was the rectal mucosa obtained separately by rectal endoscopy. All biopsies obtained were stored in cryogenic tubes containing RNALater, a stabilizing reagent preventing degradation of nucleic acids (without the need for freezing in the imaging facilities), and preserving structural morphology to enable pathology review. Biopsies were embedded in OCT wax. Slices of 0.5 microns were cut and stained with hematoxylin and eosin, in order to assess the percentage of tumor cells and identify any contamination with adjacent tissues. When necessary, microdissection was performed to enrich tumor content to the required threshold (≥50% of tumor content).

Tissue biopsies that had passed quality control were then lysed with a polytron, homogenized in a lysis buffer RLT Plus provided in the kit, and DNA was obtained with a specific affinity silica matrix column, specifically retaining the DNA, whereas RNAs and proteins were collected from the through flow. DNA was washed and eluted. The through flow containing RNAs was mixed with tri-reagent, and subsequently RNA was obtained by isopropanol precipitation. This procedure enables collection of all types of RNAs, including messenger RNAs and small microRNAs species. RNAs pelleted through centrifugation were washed will ethanol 75%, and solved in nuclease-free water. Quality control for DNAs and RNAs was performed using spectrophotometry absorbance (Nanodrop) and through electrophoresis, using lab-on-a-chip technology from Agilent Technologies.

Gene expression direct comparison of tumor tissue and normal tissue RNAs was performed using Agilent ink jet printing 8× 60k oligo-arrays and dual color technology, using standard operating procedures and reagents supplied by Agilent Technologies. A total of 100 ng of each tumor and normal tissue total RNA was used to generate double-stranded complementary DNA, using MMLV Reverse transcriptase and oligo DT primers coupled with the promoter sequence of T7 RNA. Probe labeling and linear amplification were generated using Agilent Technologies reagents and T7 RNA pol that generated labeled complementary labeled RNAs (tumor labeled with Cy5 and normal with cRNAs with Cy5). After fragmentation and purification of labeled cRNA, hybridizations were performed as dual dye swapped (direct and inversed labeling) experiments with direct co-hybridization of equal amounts of labeled tumor and normal probes. After washing drying, microarrays were read using Agilent 2000 scanner version C. After processing with Agilent Feature extractions software, data were used for direct comparison of intensities and editing of a report containing a data quality information, identity of mRNAs differentially expressed (overexpressed or underexpressed in tumor versus normal, and providing for each gene fold changes and intensities together with *p* values for each measure, type of structural abnormalities (amplifications/deletions), threshold, fold changes, and intensities).

### DDPP algorithm

The concept of DDPP algorithm is derived from the Euclidian linear hyperspace model, in which the distance between different outcomes can be defined using multiple vectors (or coordinates). The cornerstone of the Euclidian model’s application to precision oncology and to DDPP is the identification and summation of the optimal coordinates, which are defined as the mechanism-based key genes that govern sensitivity to each of the targeted medications investigated. The DDPP methodology that applies to any type of drugs is based on the following steps:

1. *Identification from the literature*, of the key genes governing the sensitivity for a given therapeutic regimen and exploring their differential expression in tumor compared to analogous normal tissue. This dramatically reduces the number of features (genes) that are considered in subsequent steps. As we observed only six instances (patients with known PFS values under treatment with everolimus), this is a crucial stage: without dramatic decrease in the number of genes, overfitting (i.e., the development of a perfect model that is specifically tailored to the given dataset used for training), is almost guaranteed. Identification of key genes involved in drug’s mechanisms of action, based on recent literature and based on the FDA US Prescribing Information (USPI).
  - *Everolimus*—key genes: *PIK3CA, PIK3CB*, *AKT1*, *MTOR*, *FKBP1A*, *RPS6KB1*, *EIF4EBP1*, *HIF1A*, *TSC1*, *TSC2*, *AKT2*, *RPTOR*, *PTEN*, *RHEB*, *MLST8*, *RICTOR*, and *VEGFA.*
  - *Axitinib*—key genes: *VEGFA*, *VEGFB*, *VEGFC, PDGFA*, *PDGFB*, *FLT1 (VEGFR1)*, *KDR (VEGFR2)*, *FLT4 (VEGFR3)*, *PDGFRA*, *PDGFRB*, *KIT*, *KITLG*, and *FIGF.*
  - *Trametinib*—key genes: *MEK1* (*MAP2K1)*, *MEK2 (MAP2K2)*, *ARAF*, *BRAF*, *RAF1*, *ERK1 (MAPK3)*, *ERK2 (MAPK1)*, *MAPK10*, *KRAS*, *HRAS*, *NRAS*, *KSR1*, and *RAP1A.*
  - *Afatinib*—key genes: EGFR, *ERBB2*, *ERBB3*, *ERBB4* and their ligands *EGF*, *TGFA*, *AREG*, *EREG*, *HBEGF*, *BTC*, *NRG1*, *NRG2*, *NRG4.*
  - *FGFR inhibitors*: *FGFR1*, *FGFR2*, *FGFR3*, *FGFR4* and the *FGF* ligands 1, 2, 3, 4, 5, etc.
  - *Anti-PD-1/PDL-1*—*k*ey genes: *PDL-1*, *PDL-2*, *PD-1*, *CTLA-4*, *CD28*, *CD80*, *CD86*, *LAG-3*, and *TLR-4*, together with specific markers of the presence of effector tumor-infiltrating immune cells: *CD8A* (cytotoxic lymphocytes T), *CD16* (natural killer cells), and *FOXP3* (T-regs cells).

2. *Selection of the patients* with available transcriptomics data and clinical outcome (PFS) under treatment with each drug available. Minimum three patients are required. Everolimus (*n* = 6); axitinib (*n* = 5);
  - Preferably, patients with non-censored PFS were selected for investigations.
  - Only two patients had censored PFS under treatment (ID 203 treated with everolimus and ID 183 treated with pembrolizumab who had exceptional PFS >60 months, and were still under treatment). We did not discard them from the analysis, but considered them de-censored.
3. *Comparing (empirically) the performance of all top-ranking subsets*: our method is based on ranking each of the selected features (genes), based on their relative association with the dependent variable (i.e., PFS). This step can be performed in multiple ways: genes can be ranked by the strength of their univariate linear regression with PFS using parametric (e.g., Pearson) or nonparametric (e.g., Spearman) correlation, or by using Cox univariate regression model for each gene, which also has the advantage of including patients with censored PFS. Each of these methods can rank all investigated genes (17 for everolimus or 13 for angiogenesis) based on their *R*^2^ and/or *p* value, when using univariate linear regression with PFS. We then propose to generate from this ranked list all subsets that include the genes by their rank. Given that the first step identifies K genes, this means checking the top 1, 2, 3, … K features sets. This is only realistic when K is a small number. If K is large, it becomes too likely the one of the many subsets that are created happen to give good correlation with the outcome by chance (the multiple testing problem) in small cohorts. As a result, this approach depends on step 1 to choose a small subset (keeping K small).
4. *Predictive model development*: in this step the chosen features are combined into a single prediction of the outcome*.*
  A common regression method is MLR. In MLR, this step is typically achieved with the step-in approach. In this approach, features are first ranked by the individual linear association with the dependent class. The most correlated feature is introduced to the model. Features are added sequentially choosing the feature (gene) that most significantly improves the accuracy of the MLR model developed with the new set of features. The process is stopped when none of the features significantly improves that MLR model. Since modeling requires estimating *k* + 1 parameters for *k* features, deciding to add the *k*th feature requires a good estimate of *k* + 2 parameters from the data. This result in an equation of the form *y*_*i*_ = *β*_0_ + *β*_1_*x*_*i*1_ + *β*_2_*x*_*i*2_+…+*β*_*k*_*x*_*ik*_ + *ϵ*, where *y*_*i*_ is the prediction for individual *i*, *x*_*i*1,_ *x*_*i*2_ … *x*_*i*k_ are the values of the first, second, and *k*th features for individual *i*, *β*_0_, *β*_1_..*β*_*k*_ are coefficients (constants) that are learned during the training of the MLR model, and *ε* is an error term. In essence, the algorithm sums *k* features to a single prediction by using a linear sum of the features, each weighted with the same *β*_1_–*β*_*k*_ coefficients, and the algorithm chooses these coefficients (as well as *β*_0_, the intercept of the model) to minimize the differences between predicted and actual outcomes.
  Another commonly used regression method is MCR, which is often more suitable for irreversible events (such as progression). This approach is very similar to MLR, differing from MLR mostly in the model that connects feature value (gene expression) with the dependent target variable (PFS): MCR works in a logarithmic and not linear space. Otherwise, features selection can be achieved the same way, considering the significance of improvement in fit between the predicted and actual outcomes (PFS). MCR builds a model that connects the outcome variable (in our case, PFS) with a very similar approach to MLR, by seeking the values of *k* + 1 parameters $\left( {\beta _{0} - \beta _{{k}}} \right)$ except that it maximizes the fit to a different equation, namely ${{y}}_{{i}} = \beta _0 \cdot {{e}}^{(\beta _1 \times _{{{i}}1} + \beta _2 + _{{{i}}2} + \ldots + \beta _{{k}} \times _{{{ik}}})}$.

We note that neither MLR nor CMR are expected to perform well in this task, considering the small number of samples that are under investigation, both methods are too likely to over fit the model to the data. As both rely on parametric linear combination for combining the features into a single method.

We propose the Digital Display Precision Predictor (DDPP) algorithm, as an alternative way for features summation that is parameter free. DDPP is different from these approaches as it empirically chooses the summation method that gives the best model, considering in the current version five basic, parameter-free summation methods: sum, mean, product, absolute product, and scalar median. It differs from the methods described above in not trying to estimate any coefficients in the summation step, making it especially suitable for small datasets, as it is less likely to over fit.

In conclusion, DDPP is unique in two levels:

- It empirically decides how many features (genes) to include in the predictors rather than stopping when the fit is no longer improved.
- It empirically decides which basic parameter-free summation method best predicts the outcome.

5. *Combined gene expression (parameter-free feature summation)*: in a previous publication^10^, we explain how we derive *F*_*i,g*_ from two color arrays. Briefly Equation (1) describe how feature i is calculated for gene *g* *.*
  1 $$F_{i,g} = \log_{2}\frac{T_{i,g}}{N_{i,g}} \cdot \log_{1.1}I_{i,g,{\mathrm{tumor}}}$$
  where *F*_*i,g*_ is used as the measurement for gene *g* in individual *i*, *T*_*i,g*_, and *N*_*i,g*_ are the measured intensity of gene *g* in individual *i* correspondingly. In order to get a single value from multiple features (genes), we combined for each individual *i* all genes values (*F*_*i,g*1_, *F*_*i,g*2_… *F*_*i,gN*_) using different approaches: (1) mean: $\overline {F_{i,g}}$; (2) sum: $\mathop {\sum}\nolimits_{x = 1}^N {F_{gx}}$; (3) median; (4) fold: $\mathop {\prod}\nolimits_{x = 1}^N {F_{gx}}$, and (5) fold_abs: $\mathop {\prod}\nolimits_{x = 1}^N {\left| {F_{gx}} \right|}$.
6. *To define the optimal* “*n*” *genes* *investigated* *we* *interrogated the correlation between gene expression and the PFS*: for each drug, a Pearson correlation test was performed between *F*_*g*_, the fold change multiplied by the intensity (of tumor and normal) of a single gene *g* (gene from the list of key genes of the drug) with the PFS for all the patients treated with the drug.
  $$\begin{array}{l}F_g = \mathrm{log2}\left( {\mathrm{fch}}\;{\mathrm{tumor}}\;{\mathrm{vs.}}\;{\mathrm{normal}} \right)\ast \mathrm{log1.1}\left( \mathrm{{tumor}} \right)\quad \quad {\mathrm{or}}\\ F_g = \mathrm{log2}\left({\mathrm{fch}}\;{\mathrm{tumor}}\;{\mathrm{vs.}}\;{\mathrm{normal}} \right)\ast \mathrm{log1.1}\left( \mathrm{{normal}} \right)\end{array}$$
  The *F*_*g*_ with the most significant correlated gene (by *p* value and correlation coefficient *r*) was driven to decision whether to continue with fold change multiplied by the intensity of the tumor or fold change multiplied by the intensity of the normal matched tissue. The key genes were then ranked based on the Pearson *p* value and correlation coefficient *r* such that the gene with the highest correlation between *F*_*g*_ and the PFS was ranked first. Then, we added single genes by the following manner: the second most ranked gene was added to the first most ranked and the *F*_*g*1,*g*2_. This resulted with vector space which contains two vectors. Similarly, the third most ranked gene was added to the second highest ranked genes, and resulted with vector space with three vectors. The vector summation continued in a similar manner until we obtain *n* different combinations of genes, while *n* is the number of genes in the set and each combination denotes a vector space with *n* vectors in it. In order to get a single value for each vector space, we tested the five different basic mathematical operations on the vectors in each vector space mentioned above.
  Then, a Pearson correlation test was performed between the calculated transcriptomic value for each vector space with the PFS of the patients treated with the drug. The Pearson correlation results were ranked again by *p* value and correlation coefficient *r*. The number of genes in the set which was the most correlated with the PFS was indicated as the optimal “*n*” coordinates. In order to assess the likelihood of getting a significant correlator by “*n*” genes, we run an analysis with 100 K random “*n*” genes and tested how the transcriptomic value derived from a vector space which includes *F*_*g*1,…*gn*_ of these genes were correlated with the PFS. Significant results were considered by a threshold of absolute *R* value of 0.9 or above, and *p* value of 0.05 and below.
  Applying any of the mathematical operations listed above resulted with a single value that combines the transcriptomic expression of all targetable genes for each patient. These values were used in order to generate the linear regression model with the PFS observed.
7. *Selecting the best correlators with PFS for each drug and computing a linear regression model to transform the best correlator into a predictor for a single drug.* Supplemental Table 3 shows the log2 fold change (intensity in tumor versus intensity in normal) multiplied by the intensity of tumor of selected eight genes for patients treated with everolimus. The expression values were used to calculate DDPP values based on fold_abs, which is absolute value of the multiplication of all the gene’s values in each patient. The DDPP values are shown in Supplemental Table 4. Then, we used the DDPP values and the real PFS values as shown in Supplemental Table 5 for Pearson correlation with 95% confidence interval analysis and linear regression model analysis (which provided the linear equation). The results of these analyzes are shown in Supplemental Fig. 3.
8. Linear regression equations developed to link gene expression with clinical outcome under specific treatments:
  Everolimus—Equation 1: *Y* = 1.499e−13*X* + 3.134, where *X* = the absolute value of the fold of log2(fold change tumor versus normal) multiplied by log1.1 (Intensity_Tumor) of each value for each of the eight genes (*AKT2*, *TSC1*, *FKB-12*, *TSC2*, *RPTOR*, *RHEB*, *PIK3CA*, and *PIK3CB*) and *Y* = PFS in months.
  Axitinib—Equation 2: *Y* = 2.014e−02*X* + 4.36, where *X* = the sum of log2(fold change tumor versus normal) multiplied by log1.1 (Intensity_Normal) of each value for each of the two genes (*KIT-KITLG)*, and *Y* = PFS in months.
  Trametinib—Equation 3: *Y* = −6.872e−15*X* + 7.745, where *X* = the fold of log2(fold change tumor versus normal) multiplied by log1.1 (Intensity_Tumor) of each values for each of the nine genes (*ERK2*, *ARAF*, *CRAF*, *MEK1*, *MEK2*, *HRAS*, *ERK1*, *MAPK10*, and *KSR1)*, and *Y* = PFS in months.
  Afatinib—Equation 4: *Y* = −4.558e−02*X* + 2.549, where *X* = the sum of log2(fold change tumor versus normal) multiplied by log1.1 (Intensity_Tumor) of each value for each of the two genes (*NRG4* and *NRG2*), and *Y* = PFS in months.
  FGFR inhibitors—Equation 5: *Y* = −5.273e−02*X* + 5.135, where *X* = the sum of log2(fold change tumor versus normal) multiplied by log1.1 (Intensity_Normal) of each value for each of the five genes (*FGF10*, *FGF16*, *FGF5*, *FGF2*, and *FGF13)*, and *Y* = PFS in months.
  Anti-PD-1—Equation 6: *Y* = 7.856e−10*X* – 1.583, where *X* = the value of the fold of log2(fold change tumor versus normal) multiplied by log1.1 (Intensity_Normal) of each value for each of the six genes (*TLR-4*, *PDL-2*, *PDL-1*, *CD16 (NK)*, *CTLA-4*, and *CD28)*, and *Y* = PFS in months.

### Reporting summary

Further information on research design is available in the Nature Research Reporting Summary linked to this article.

## Supplementary information

Supplemental data

REPORTING SUMMARY

## Data Availability

Expanded clinical and biological information for each patient is available in the data file Patient_demographic_clinical_data.csv^29^ which expands on the information presented in Table 1. The data file DPP_WINTHER_Expression_Data.csv^29^ supports Figs. 1–3, Supplementary Tables 1, 3–5, and Supplementary Figs. 1–3. The transcriptomic expression data are available from Gene Expression Omnibus: https://identifiers.org/geo:GSE168621 (ref. ^30^). The data generated and analyzed during this study are described in the following data record: 10.6084/m9.figshare.14166731 (ref. ^29^).

## Code availability

Codes are available upon request to the corresponding author.
