# Supplementary material for: Digital Display Precision Predictor: the prototype of a global biomarker model to guide treatments with targeted therapy and predict progression-free survival
Source: NPJ Precis Oncol. 2021 Apr 28;5:33. doi: 10.1038/s41698-021-00171-6 (PMC8080819; doi:10.1038/s41698-021-00171-6)
Supplement: Supplementary file 1 — Supplemental data [file 41698_2021_171_MOESM1_ESM.pdf]

Digital Display Precision Predictor: the prototype of a global biomarker model to guide treatments with targeted therapy and predict progression-free survival

Lazar Vladimir[1*], Magidi Shai[1*], Girard Nicolas[2], Savignoni Alexia[2], Martini Jean-François[3], Massimini Giorgio[4], Bresson Catherine[1], Berger Raanan[5], Onn Amir[5], Raynaud Jacques[6], Wunder Fanny[1], Berindan-Neagoe Ioana[7], Sekacheva Marina[8], Braña Irene[9], Tabernero Josep[9], Felip Enriqueta[9], Porgador Angel[10], Kleinman Claudia[11], Batist Gerald[11], Solomon Benjamin[12], Tsimberidou Apostolia Maria[13], Soria Jean-Charles[14], Rubin Eitan[10*], Kurzrock Razelle[15*], Schilsky Richard L.[16*]

Supplementary notes:

Alternative methods for the identification of individual contribution of the 17 key genes to predict PFS under everolimus treatment for everolimus:

Spearman non-parametric model versus Pearson parametric model: Spearman correlations between differential gene expression and PFS for each of the 17 genes involved in the everolimus pathway were (sorted by r^2): *PIK3CB* (r=-0.94, *p=0.017*), *TSC2* (r=0.83, *p=0.058*), *RHEB* (r=-0.83, *p=0.058*), *AKT2* (r=0.71, *p=0.136*), *PIK3CA* (r=0.54, *p=0.297*), MLST8 (r=-0.54, *p=0.297*), S6K1 (r=0.43, *p=0.419*), *RPTOR* (r=0.43, *p=0.419*), *FKB-12* (r=-0.31, *p= 0.564*), *TSC1* (r=0.31, *p=0.564*), *PTEN* (r=-0.26, *p=0.658*), *AKT1* (r=-0.2, *p=0.714*), *MTOR* (r=0.2, *p=0.714*), *HIF1* (r=0.14, *p=0.803*), *4E-BP1* (r=-0.09, *p=0.919*), *RICTOR* (r=-0.03, *p=1*) and *VEGFA* (r=-0.03, *p=1*). We further explored the combined differential expression in tumor versus normal tissues of the most contributive key genes involved in the everolimus pathway. For each of the correlations with PFS, we built a vectorial summation using a 'step-in' method, starting with *PIK3CB*, which was identified as the most contributive gene according to Spearman correlation, and adding successively a single gene at each step in the order of their significance: *PIK3CB-TSC2; PIK3CB-TSC2-RHEB*; then *PIK3CB-TSC2-RHEB-AKT2* and so forth, obtaining in total 17 different summations. Each summation was correlated with the observed PFS. The optimal performance was obtained by the summation of the 10 most contributive genes: *PIK3CB, TSC2, RHEB, AKT2, PIK3CA, MLST8, S6K1, RPTOR, FKB-12* and *TSC1*, which yields the most significant Spearman correlation with the observed PFS among the different 17 vector summation possibilities (r=-0.94, *p=0.017*). The DDPP linear regression model using the genes ranked by Spearman correlation with PFS is: Y= -1.645e-16X + 3.642, where X = the fold value of the log2(Fold change tumor *versus* normal) multiplies by log1.1(Intensity_Tumor) of each value for each of the 10 genes, and Y = PFS in months. A comparison with DDPP

Pearson-based model significantly outperformed Spearman-based models, with a 3-fold smaller p value. Therefore, Pearson linear regression was selected.

Cox univariate regression model: Using Cox univariate regression, the 17 genes identified as everolimus-related ranked by increasing p value were: *PIK3CB* (*p=0.023*), *AKT2* (*p=0.075*), *TSC2* (*p=0.081*), *RPTOR* (*p=0.109*), *RHEB* (*p=0.117*), *FKBP1A* (*p=0.235*), *PIK3CA* (*p=0.245*), *MLST8* (*p=0.262*), *TSC1* (*p=0.267*), *HIF1A* (*p=0.431*), *AKT1* (*p=0.476*), *RICTOR* (*p=0.601*), *PTEN* (p=0.606), *RPS6KB1* (p=0.607), *VEGFA* (p=0.705), *EIF4EBP1* (p=0.812) and *MTOR* (p=0.995). The optimal performance in predicting PFS was obtained with the first 9 genes: *PIK3CB, AKT2, TSC2, RPTOR, RHEB, FKB-12, PIK3CA, MLST8* and *TSC1* (correlation between predicted and actual PFS: r=0.99, *p=5.56E-05*). The regression model for the correlation with PFS is: Y= 3.488e-15X – 3.198, where X= the absolute value of the fold of log2(Fold change tumor versus normal) multiplied by log1.1 (Intensity_Tumor) of each value for each of the 9 genes, and Y = PFS in months. A direct comparison of the DDPP model developed with Pearson linear regression ranking and developed with Cox univariate regression for ranking revealed that identical genes were selected (with the exception of *MLST8*) and generated very similar predictors.

These results suggest that despite a small cohort, DDPP enabled the identification of a stable predictor of the PFS.

Cox multivariate analysis: Cox multivariate regression method identified a subgroup of 4 genes that had the highest significance for the association with PFS categories: *AKT2, TSC2, RPTOR* and *PIK3CB* (Concordance =1), Rsquare=0.888, Likelihood ratio test=13.16 on 4df (p=0.01), Wald test=0 on 4df (p=1) and Score (logrank) test =9.05 on 4 df (p=0.06). However, the DDPP predictor of PFS for this limited subset of genes (*AKT2, TSC2, RPTOR* and *PIK3CB*) was: r=0.77 and *p=0.075*. The regression model for the correlation with PFS is: Y=6.890e-07X + 2.658, where X= the absolute value of the fold of log2(Fold change tumor versus normal) multiplied by log1.1 (Intensity_Tumor) of each value for each of the 4 genes, and Y = PFS in months.

Multiple linear regression (MLR): The MLR method selected 4 genes that best explained the PFS: *AKT2, RICTOR, TSC1* and *MLST8*. The MLR model for the 4 genes achieved r=1, r-squared 0.999; Adjusted r-squared 0.997 and predicted r-squared 0.774. The root mean square error was 1.23 with a coefficient of variability 8.722, a mean square error of 1.513 and a mean absolute error of 0.431. The highest significant DDPP predictor of PFS for this limited subset of genes (*AKT2, RICTOR, TSC1* and *MLST8*) was: r=-0.86 and *p=0.026*. The regression model for the correlation with PFS is: Y= -1.599e-06X + 1.889, where X= the fold of log2(Fold change tumor versus normal) multiplied by log1.1

(Intensity_Tumor) of each value for each of the 4 genes, and Y = PFS in months. In conclusion, the multiple linear regression method could not be used to generate a DDPP predictor, because of the error model that stops the selection of the genes when the model reaches r=1.

Influence of the correlation of the co-expression of key genes for everolimus: To investigate whether the genes are naturally correlated with each other, and to explore influence of these correlations on the selection of optimal genes, we generated the matrix of Pearson correlations of co-expression of the everolimus 17 key genes displayed in Supplemental Table 1.

R	PIK3CA	PIK3CB	AKT1	MTOR	FKBP1A	RPS6KB1	EIF4EBP1	HIF1A	TSC1	TSC2	AKT2	RPTOR	PTEN	RHEB	MLST8	RICTOR	VEGFA
PIK3CA	1,00	-0,02	-0,67	0,21	-0,68	0,44	-0,73	-0,58	0,55	0,28	0,92	0,90	0,16	-0,67	-0,51	0,93	0,79
PIK3CB	-0,02	1,00	-0,30	0,49	-0,18	-0,43	-0,47	-0,65	0,22	-0,47	-0,11	-0,29	0,45	0,52	0,11	0,15	0,50
AKT1	-0,67	-0,30	1,00	-0,19	0,84	0,01	0,94	0,66	-0,48	0,05	-0,68	-0,45	-0,72	0,31	0,65	-0,57	-0,64
MTOR	0,21	0,49	-0,19	1,00	-0,15	0,18	-0,44	-0,16	0,82	0,52	0,38	0,33	-0,28	0,48	-0,48	0,42	0,70
FKBP1A	-0,68	-0,18	0,84	-0,15	1,00	0,26	0,72	0,41	-0,61	-0,08	-0,75	-0,53	-0,47	0,54	0,41	-0,45	-0,62
RPS6KB1	0,44	-0,43	0,01	0,18	0,26	1,00	-0,16	-0,06	0,13	0,53	0,41	0,60	-0,35	-0,14	-0,54	0,60	0,22
EIF4EBP1	-0,73	-0,47	0,94	-0,44	0,72	-0,16	1,00	0,78	-0,59	-0,02	-0,70	-0,52	-0,59	0,14	0,68	-0,75	-0,82
HIF1A	-0,58	-0,65	0,66	-0,16	0,41	-0,06	0,78	1,00	-0,12	0,48	-0,33	-0,21	-0,67	0,10	0,16	-0,67	-0,67
TSC1	0,55	0,22	-0,48	0,82	-0,61	0,13	-0,59	-0,12	1,00	0,67	0,77	0,67	-0,18	-0,04	-0,63	0,55	0,79
TSC2	0,28	-0,47	0,05	0,52	-0,08	0,53	-0,02	0,48	0,67	1,00	0,56	0,66	-0,69	-0,10	-0,58	0,29	0,26
AKT2	0,92	-0,11	-0,68	0,38	-0,75	0,41	-0,70	-0,33	0,77	0,56	1,00	0,95	0,02	-0,58	-0,69	0,82	0,78
RPTOR	0,90	-0,29	-0,45	0,33	-0,53	0,60	-0,52	-0,21	0,67	0,66	0,95	1,00	-0,21	-0,62	-0,61	0,84	0,68
PTEN	0,16	0,45	-0,72	-0,28	-0,47	-0,35	-0,59	-0,67	-0,18	-0,69	0,02	-0,21	1,00	-0,07	-0,14	0,08	0,13
RHEB	-0,67	0,52	0,31	0,48	0,54	-0,14	0,14	0,10	-0,04	-0,10	-0,58	-0,62	-0,07	1,00	0,03	-0,38	-0,15
MLST8	-0,51	0,11	0,65	-0,48	0,41	-0,54	0,68	0,16	-0,63	-0,58	-0,69	-0,61	-0,14	0,03	1,00	-0,54	-0,49
RICTOR	0,93	0,15	-0,57	0,42	-0,45	0,60	-0,75	-0,67	0,55	0,29	0,82	0,84	0,08	-0,38	-0,54	1,00	0,86
VEGFA	0,79	0,50	-0,64	0,70	-0,62	0,22	-0,82	-0,67	0,79	0,26	0,78	0,68	0,13	-0,15	-0,49	0,86	1,00

It should be noted that the differential tumor *versus* normal expression of the 17 key genes related to everolimus is not an independent variable. The most significant correlation (r ≥0.9) of their co-expression is observed for*: i)* co-expression of *PIK3CA AKT2, RPTOR* and *RICTOR* and *ii)* co-expression of *EIF4BP1* and *AKT1*.

We concluded that this matrix did not influence in any way the DDPP algorithm. Indeed, the order of genes obtained through Pearson (P) or Cox (C) univariate models were totally different. However, the order of genes does not seem to be a function of the correlation between their co-expressions.

P: *AKT2, TSC1, FKB-12, TSC2, RPTOR, RHEB, PIK3CA, PIK3CB*

C: *PIK3CB, AKT2, TSC2, RPTOR, RHEB, FKB-12, PIK3CA, MLST, TSC1*

Whilst DDPP summation uses the order of genes to perform the "step-in" combinations, the final result does not depend on the order of the genes itself (as long as the set of genes is identical), which is the case above (with the exception of *MLST*).

Supplemental Table 1: Everolimus mechanism of action and the key genes for everolimus pathway

Everolimus is an inhibitor of mammalian target of rapamycin (mTOR), a serine-threonine kinase, downstream of the PI3K/AKT pathway. The mTOR pathway is dysregulated in several human cancers. Everolimus binds to an intracellular protein, FKBP-12, resulting in the formation of an inhibitory complex (mTORC1) and thus inhibition of mTOR kinase activity. Everolimus reduces the activity of S6 ribosomal protein kinase (S6K1) and eukaryotic elongation factor 4E-binding protein (4E-BP1), downstream effectors of mTOR, involved in protein synthesis. In addition, everolimus inhibits the expression of hypoxia-inducible factor (e.g., HIF-1) and reduces the expression of vascular endothelial growth factor (VEGF). Inhibition of mTOR by everolimus. Everolimus has been shown to reduce cell proliferation, angiogenesis, and glucose uptake.		
Gene	Symbol	Role
Phosphatidyl-inositol-Bisphosphate Kinase Catalytic Subunit Alpha	PIK3CA	Generates phosphatidylinositol 3,4,5-trisphosphate (PIP3). Involved in the activation of AKT1 upon stimulation by receptor tyrosine kinases ligands such as EGF, insulin, IGF1, VEGFA and PDGF. Essential in endothelial cell migration during vascular development through VEGFA signaling, possibly by regulating RhoA activity.
Phosphatidyl-inositol-Bisphosphate Kinase Catalytic Subunit Beta	PIK3CB	Generates PIP3. Involved in the activation of AKT1 upon stimulation by G-protein coupled receptors (GPCRs) ligands such as CXCL12, kinases. Plays a role in platelet activation signaling triggered by GPCRs, alpha-IIb/beta-3 integrins (ITGA2B/ ITGB3) and ITAM.
AKT Serine Threonine Kinase 1	AKT1	Plays a key in regulating cell survival, insulin signaling, angiogenesis and tumor formation. AKT1 is a downstream mediator of the PI 3-K pathway, which results in the recruitment of Akt to the plasma membrane.
AKT Serine Threonine Kinase 2	AKT2	Plays a key role in regulating cell survival, insulin signaling, angiogenesis and tumor formation. AKT2 is a downstream mediator of the PI 3-K pathway, which results in the recruitment of Akt to the plasma membrane.
Phosphatase And Tensin Homolog	PTEN	Tumor suppressor. It negatively regulates intracellular levels of phosphatidylinositol-3,4,5-trisphosphate in cells and functions as a tumor suppressor by negatively regulating AKT/PKB signaling pathway.
Tuberous sclerosis Complex Subunit 1	TSC1	Inhibits the nutrient-mediated or growth factor-stimulated phosphorylation of S6K1 and EIF4EBP1 by negatively regulating mTORC1 signaling. Seems not to be required for TSC2 GAP activity towards RHEB. Involved in microtubule-mediated protein transport.
Tuberous sclerosis Complex Subunit 2	TSC2	Inhibits the nutrient-mediated or growth factor-stimulated phosphorylation of S6K1 and EIF4EBP1 by negatively regulating mTORC1 signaling. Acts as a GTPase-activating protein (GAP) for the small GTPase RHEB, a direct activator of the protein kinase activity of mTORC1.
Ras Homolog, MTORC1 Binding	RHEB	Vital in regulation of growth and cell cycle progression due to its role in the insulin/TOR/S6Ksignaling pathway. Activates the protein kinase activity of mTORC1, and thereby plays a role in the regulation of apoptosis. Stimulates the phosphorylation of S6K1 and EIF4EBP1 through activation of mTORC1 signaling. Has low intrinsic GTPase activity.
FKBP Prolyl Isomerase 1A	FKB-12	Plays a role in immunoregulation and basic cellular processes involving protein folding and trafficking. Binds the immunosuppressants FK506 and rapamycin. It interacts with several intracellular signal transduction proteins including type I TGF-beta receptor.
Mechanistic Target Of Rapamycin Kinase	MTOR	Target for the cell-cycle arrest and immunosuppressive effects of the FKBP12-rapamycin complex. Functions as part of 2 structurally and functionally distinct signaling complexes mTORC1 and mTORC2. Activation of MTORC1 trigger phosphorylation of EIF4EBP1 and release of its inhibition toward the elongation initiation factor 4E (eiF4E). Phosphorylates and activates RPS6KB1 that promotes protein synthesis.
MTOR Associated Protein, LST8 Homolog	MLST8	Subunit of both mTORC1 and mTORC2. Within mTORC1, LST8 interacts directly with MTOR and enhances its kinase activity. In nutrient-poor conditions, stabilizes the MTOR-RPTOR interaction and favors RPTOR-mediated inhibition of MTOR activity. mTORC2 is also activated by growth factors, but seems to be nutrient-insensitive.
Regulatory Associated Protein Of MTOR Complex 1	RPTOR	Forms a stoichiometric complex with the mTOR kinase (MTORC1), and also associates with eukaryotic initiation factor 4E-binding protein-1 and ribosomal protein S6 kinase. The protein positively regulates the downstream effector ribosomal protein S6 kinase, and negatively regulates the mTOR kinase.
RPTOR Independent Companion of MTOR Complex 2	RICTOR	Subunit of mTORC2: regulates cell growth and survival in response to hormonal signals. mTORC2 is activated by growth factors, but, in contrast to mTORC1, is nutrient-insensitive. mTORC2 seems to function upstream of Rho GTPases to regulate the actin cytoskeleton, probably by activating one or more Rho-type guanine nucleotide exchange factors. mTORC2 promotes the serum-induced formation of stress-fibers or F-actin.
Ribosomal Protein S6 Kinase B1	S6K1	Acts downstream of mTOR signaling in response to growth factors and nutrients to promote cell proliferation, cell growth and cell cycle progression. Regulates protein synthesis through phosphorylation of EIF4B, RPS6 and EEF2K, and contributes to cell survival by repressing the pro-apoptotic function of BAD.
Eukaryotic Translation Initiation Factor 4E Binding Protein 1	4EBP1	Translation repressor protein. Directly interacts with eukaryotic translation initiation factor 4E (eIF4E), which is a limiting component of the multi-subunit complex that recruits 40S ribosomal subunits to the 5' end of mRNAs.
Hypoxia Inducible Factor 1 Subunit Alpha	HIF1	Master regulator of cellular and systemic homeostatic response to hypoxia by activating transcription of many genes, including those involved in energy metabolism, angiogenesis, apoptosis, oxygen delivery or facilitate metabolic adaptation to hypoxia. Everolimus inhibits the expression of hypoxia-inducible factor (e.g., HIF-1).
Vascular Endothelial Growth Factor A	VEGFA	Growth factor active in angiogenesis, vasculogenesis and endothelial cell growth. Induces endothelial cell proliferation, promotes cell migration, inhibits apoptosis and induces permeabilization of blood vessels. Binds to the FLT1/VEGFR1 and KDR/VEGFR2 receptors. Everolimus reduces the expression of VEGFA and reduces cell proliferation, angiogenesis, and glucose uptake.

Supplemental Table 2: Description and rationale for the selection of key genes of immune blockade [5]

<table>
<tr><th>Usual Name</th><th>Official Names</th><th>Role in the negative immune-blockade</th></tr>
<tr><td colspan="3">The T lymphocytes (LyT) that infiltrate the tumor (TILS) recognize the presented tumor neo-antigens. The neo-antigens are recognized as “non-self” as they are modified proteins because of mutations. The clone of LyT that recognizes specifically the neo-antigen is activated and proliferates. The recruitment of activated LyT that recognize specifically the tumor is a complex process that involves different antigen presentation mechanisms. Professional Antigen Presenting Cells (APC) present the neo-antigen associated to the major histocompatibility complex II (CMH2) recognized by LyT CD4+ that differentiate in LyT Helper 1 (Ly Th1) and Helper 2 (Ly Th2). Ly Th1 are key in recruitment of naïve LyT CD8+ and induce their activation. Lymphocytes T Cytotoxic (CD8+) and Natural Killer lymphocytes (NK) also recognize the neo-antigen restricted to CMH1 (Histocompatibility complex 1) and are subsequently activated, and can destroy directly the tumor cells presenting the neo-antigen. The process of recruitment and activation of cytotoxic lymphocytes T CD8 is controlled by different mechanisms of the negative blockade.</td></tr>
<tr><td>PD-1
PD-L1
PD-L2</td><td>PDCD1
CD274
PDCD1LG2</td><td rowspan="2">PDL-1 and PDL-2 bind to PD-1 and directly inhibit the T receptor. CTLA-4 has a high affinity and avidity for BF1 and BF2 ligands that binds to the co-stimulatory molecule CD28. In this competitive manner, CTLA-4 blocks CD28 and has a negative blockade effect. PD-1 and CTLA-4 are highly expressed on TILs in metastatic melanoma, NSCLC, UBC, and squamous cell carcinoma of the head and neck. PD-1 and CTLA-4 modulate effector T cell activation, proliferation, and function through distinct, complementary mechanisms. The expression of PD-1 and CTLA-4 on tumor-infiltrating T cell populations contributes to suppression and immunological escape. In vivo studies have shown that tumor-infiltrating lymphocytes and not peripheral T cells have been shown to be the major contributor to tumor control following anti-PDL-1 + anti-CTLA-4 mAb therapy.</td></tr>
<tr><td>CTLA4
B7-1
B7-2
CD28</td><td>CTLA4
CD80
CD86
CD28</td></tr>
<tr><td>LAG-3</td><td>LAG3</td><td>LAG-3 delivers inhibitory signals upon binding to ligands, such as FGL1 (responsible for LAG-3 T-cell inhibitory function). Following TCR engagement, LAG-3 associates with CD3-TCR in the immunological synapse and directly inhibits T-cell activation (may inhibit antigen-specific T-cell activation in synergy with PDCD1/PD-1, possibly by acting as a co-receptor for PDCD1/PD-1). LAG-3 negatively regulates the proliferation, activation, effector function and homeostasis of both CD8(+) and CD4(+) T-cells. Also mediates immune tolerance. LAG-3 is constitutively expressed on a subset of regulatory T-cells (Tregs) and contributes to their suppressive function.</td></tr>
<tr><td>TLR-</td><td>TLR4</td><td>TLR-4 overexpression and activation by LPS activates MAPK and NF-κB pathways, implicating cell-autonomous TLR-4 signaling in regulation of carcinogenesis, in particular, through increased proliferation of tumor cells, apoptosis inhibition and metastasis. TLR-4 signaling in immune and inflammatory cells of tumor microenvironment may lead to production of pro-inflammatory cytokines (TNF, IL-1β, IL-6, IL-18, etc.), immunosuppressive cytokines (IL-10, TGF-β, etc.) and angiogenic mediators (VEGF, EGF, TGF-β).</td></tr>
<tr><td>CD8</td><td>CD8A</td><td>The level of infiltration of the tumor by Cytotoxic lymphocytes T CD8 (LyTc) can be assessed by investigating the specific marker CD8.</td></tr>
<tr><td>CD16</td><td>FCGR3</td><td>The level of infiltration of the tumor by Natural Killers cells (NK) can be assessed by investigating the specific marker is CD16.</td></tr>
<tr><td>FOXP3</td><td>FOXP3</td><td>The level of infiltration of the tumor by a specific population of Lymphocytes T called regulatory (T-regs) can be assessed by investigating the specific marker FOXP3.</td></tr>
</table>

Supplemental Table 3: Log2 fold change (tumor vs. normal) multiplied by the intensity in tumor for the 8 genes indicated in the correlator for 6 patients treated with everolimus

	X090	X227	X117	X006	X148	X203
AKT2	35.50842	-84.2814	43.13603	-64.607	149.7651	218.7834
TSC1	20.71974	35.45727	12.206	-94.1848	35.15227	152.7379
FKBP1A	-3.02155	78.28654	-3.59654	30.07732	34.53388	-44.5219
TSC2	-18.7177	60.33901	-10.8945	-9.15613	104.6628	105.2716
RPTOR	24.4181	-1.0124	17.78346	-1.34029	64.43769	57.76267
RHEB	-9.2125	40.9876	8.389341	-14.8667	-15.3537	-20.299
PIK3CA	37.88505	-51.6498	25.87614	-28.0215	66.83337	53.36838
PIK3CB	-21.4254	-28.4227	-30.3664	-38.3868	-46.9602	-38.662

Supplemental Table 4: Fold absolute DDPP values for the 8 genes indicated in the correlator for the 6 patients treated with everolimus

	X090	X227	X117	X006	X148	X203
DDPP values	7.6E+09	8.6E+11	2.42E+09	3.59E+10	5.91E+13	3.79E+14

Supplemental Table 5: PFS and DDPP values of the 6 patients treated with everolimus

ID	PFS values	DDPP values
X090	1.3	7597718579
X227	1.7	8.59924E+11
X117	1.9	2418481766
X006	8.1	35916904118
X148	11.6	5.90849E+13
X203	60	3.78909E+14

Supplemental Figure 1.

DDPP intensity plots and correlation with PFS for patients treated with axitinib

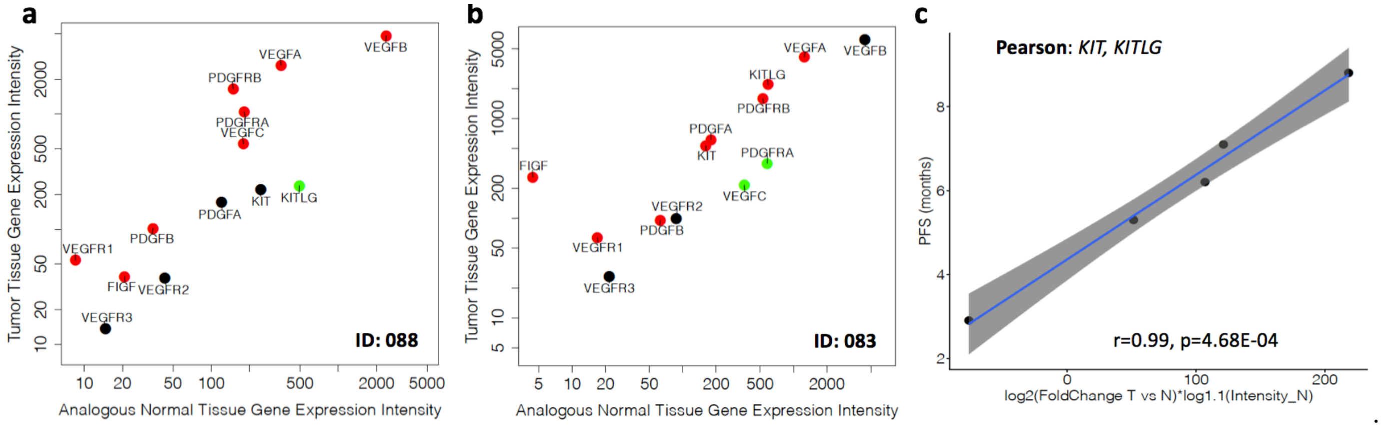

Examples of DDPP profiles for two patients treated with axitinib in monotherapy, with different outcomes. Data source WINTHER trial [11].

a: ID 088**,** NSCLC, PFS=2.9 months in 2nd therapy line

b: ID 083, HN, PFS=8.8 months in 5th therapy line.

Y axis: intensity of the expression in tumors;

X axis: intensity of the expression in normal matched tissue.

c: Plots representing the Pearson correlation of the 2 gene-predictor *(KIT, KITLG)* with the PFS of the 5 patients treated with axitinib as monotherapy (one patient NSCLC and four patients HN)

X axis: sum value of log2 based fold changes tumor *versus* normal multiplied by the log.1.1 based intensity values of the normal for each of the 2 genes selected; **Y axis**: PFS under treatment in months.

Supplemental Figure 2.

DDPP profiles and correlations with PFS for patients treated with trametinib, afatinib and experimental FGFR inhibitors

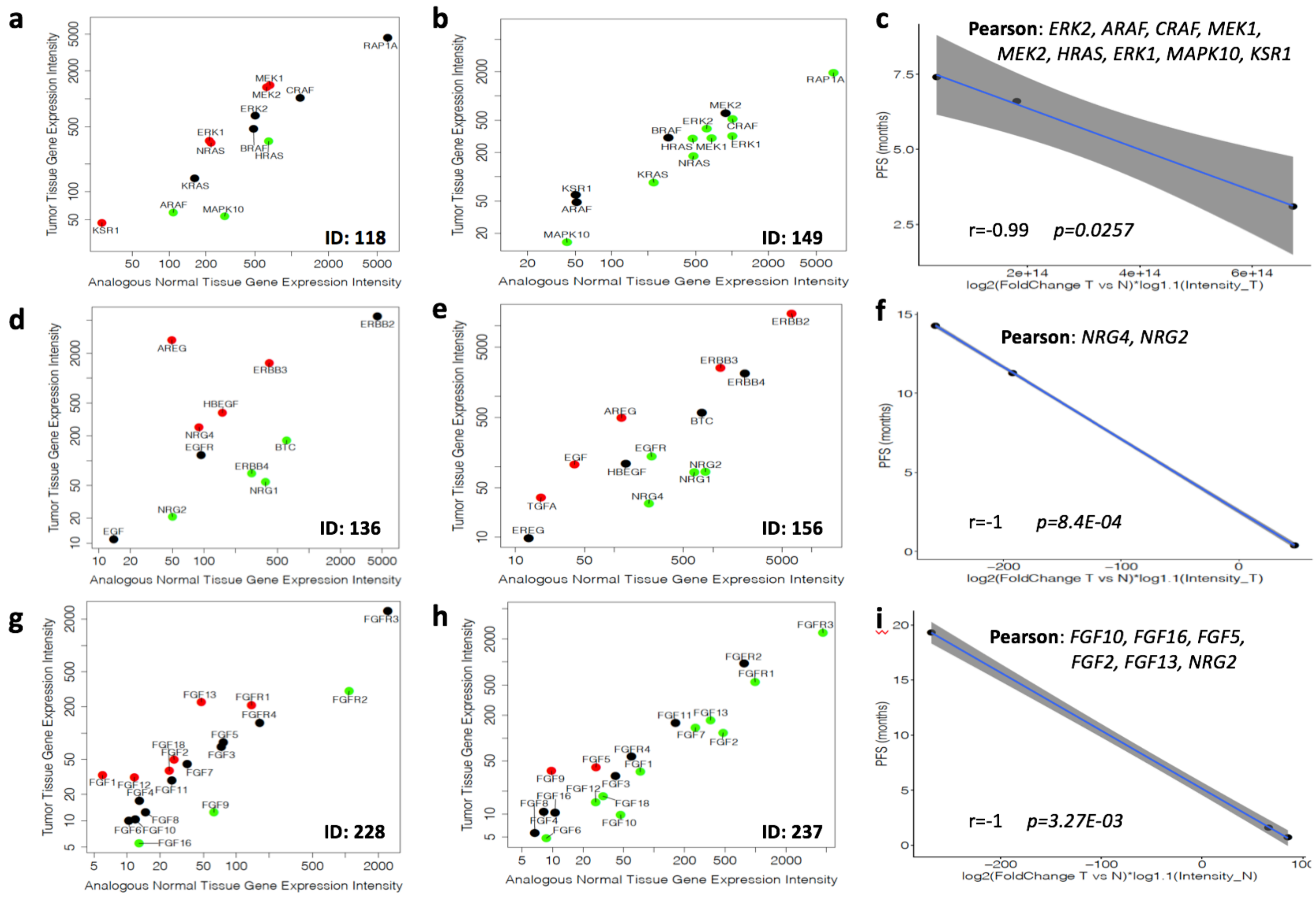

Examples of DDPP profiles for two patients treated in monotherapy with trametinib ***(a, b, c)****, afatinib* ***(d, e, f)*** *and FGFR inhibitors* ***(g, h, i),*** *with different outcomes. Data source WINTHER trial [11].*

a: ID 118**,** NSCLC, PFS=3.1 months in 4th therapy line**; b:** ID 149, Rectum carcinoma, PFS=7.4 months in 6th therapy line. **d:** ID 136, NSCLC, PFS=0.4 months in 4th therapy line; **e:** ID 156, NSCLC, PFS=14.3 months in 3rd therapy line **g:** ID 228, Colon carcinoma, PFS=0.7 months in 6th therapy line; **h:** ID 237, Head and Neck carcinoma, PFS=19.3 months in 7th therapy line. **Y axis**: intensity of the expression in tumors; **X axis**: intensity of the expression in normal matched tissue. **c:** Plot representing the Pearson correlation of the 9 gene-predictor *ERK2, ARAF, CRAF, MEK1, MEK2, HRAS, ERK1, MAPK10 and KSR1)* with the PFS of the 3 patients treated with trametinib as monotherapy **X axis:** fold value of log2 based fold changes tumor *versus* normal multiplied by the log.1.1 based intensity values of the tumor for each of the 9 genes selected **Y axis:** PFS under treatment in months. **f:** Plot representing the Pearson correlation of the 2 gene-predictor *NRG4 and NRG2,* with the PFS of the 3 patients treated with afatinib; **X axis:** the sum of log2(Fold change tumor *versus* normal) multiplied by log1.1 (Intensity_Tumor) of each values for each of the 2 genes; **Y axis** = PFS in months; **i:** Plot representing the Pearson correlation of the 5 gene-predictor *FGF10, FGF16, FGF5, FGF2 and FGF13*); **X axis**: the sum of log2(Fold change tumor *versus* normal) multiplied by log1.1 (Intensity_Normal) of each values for each of the 5 genes; **Y axis**: PFS in months.

Supplemental Figure 3:

Pearson correlation and linear regression analysis for the PFS values and DDPP values for the 6 patients treated with everolimus

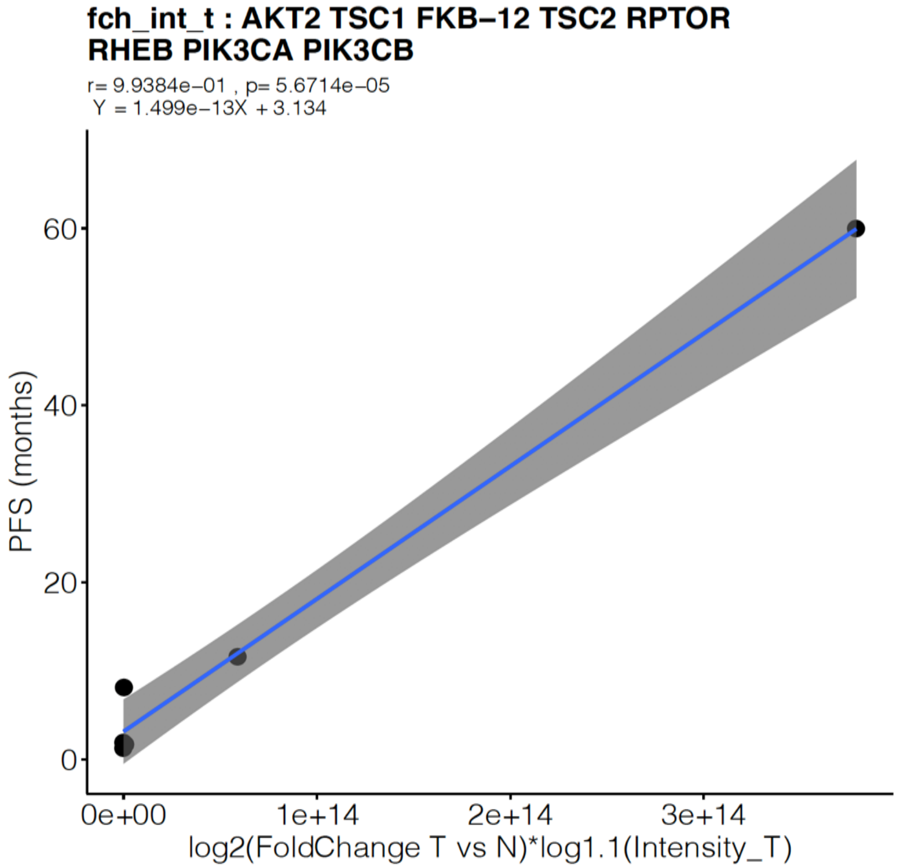

The subtitle indicates the Pearson correlation coefficient and p-value and the linear regression equation; **X axis**: the fold absolute of log2(Fold change tumor *versus* normal) multiplied by log1.1 (Intensity_Tumor) of each values for each of the 8 genes; **Y axis**: PFS in months.
